# Evidence from Meta-Analyses of the Facial Width-to-Height Ratio as an Evolved Cue of Threat

**DOI:** 10.1371/journal.pone.0132726

**Published:** 2015-07-16

**Authors:** Shawn N. Geniole, Thomas F. Denson, Barnaby J. Dixson, Justin M. Carré, Cheryl M. McCormick

**Affiliations:** 1 Psychology Department, Brock University, St. Catharines, Ontario, Canada; 2 School of Psychology, University of New South Wales, Sydney, New South Wales, Australia; 3 School of Psychology, the University of Queensland, St. Lucia 4072, Brisbane, Queensland, Australia; 4 Evolution and Ecology Research Centre, School of Biological, Earth and Environmental Sciences, University of New South Wales, Sydney, New South Wales, Australia; 5 Department of Psychology, Nipissing University, North Bay, Ontario, Canada; 6 Centre for Neuroscience, Brock University, St. Catharines, Ontario, Canada; Macquarie University, AUSTRALIA

## Abstract

The facial width-to-height ratio (FWHR) is the width of the face divided by the height of the upper face. There is mixed evidence for the hypothesis that the FWHR is a cue of threat and dominance in the human face. We conducted a systematic review and meta-analyses of all peer-reviewed studies (and 2 unpublished studies) to estimate the magnitude of the sex difference in the FWHR, and the magnitude of the relationship between the FWHR and threatening and dominant behaviours and perceptions. Studies were eligible for inclusion if the authors reported an analysis involving the FWHR. Our analyses revealed that the FWHR was larger in men than in women ($\bar{d}$ = .11, *n* = 10,853), cued judgements of masculinity in men ($\bar{r}$ = .35, *n of faces* = 487; *n of observers* = 339), and was related to body mass index ($\bar{r}$ = .31, *n* = 2,506). Further, the FWHR predicted both threat behaviour in men ($\bar{r}$ = .16, *n* = 4,603) and dominance behaviour in both sexes ($\bar{r}$ = .12, *n* = 948) across a variety of indices. Individuals with larger FWHRs were judged by observers as more threatening ($\bar{r}$ = .46, *n of faces* = 1,691; *n of observers* = 2,076) and more dominant ($\bar{r}$ = .20, *n of faces* = 603; *n of observers* = 236) than those with smaller FWHRs. Individuals with larger FWHRs were also judged as less attractive ($\bar{r}$ = -.26, *n of faces* = 721; *n of observers* = 335), especially when women made the judgements. These findings provide some support for the hypothesis that the FWHR is part of an evolved cueing system of intra-sexual threat and dominance in men. A limitation of the meta-analyses on perceptions of threat and dominance were the low number of stimuli involving female and older adult faces.

## Introduction

Perceptual and sensory systems have evolved to detect threat [1]. These systems are tuned to cues of formidability and aggressiveness in conspecifics, allowing for appropriate submissive or attack behaviours depending on the information conveyed by the cues [2–4]. The rapid communication of rank, dominance, and fighting ability may curtail the escalation of agonistic encounters; there is much evidence that agonistic contests are settled more quickly and are less likely to be lethal when animals have visual exposure to their opponent before engaging in a contest than when they do not (e.g., in cichlids, *Cichlidae*; green swordtails, *Xiphophorus hellerii;* rainbow trout, *Oncorhynchus mykiss*; pigs, *Sus scrofa*; hamsters, *Mesocricetus brandti* [4]). Although visual assessments likely depend on multiple cues of varying complexity [4], selection should favour conspicuous cues that are rapidly processed [5].

In humans, the visual system is highly sensitive to, and quick to process, cues in the face such as identity, gender, age, and emotional expression [6], which guide social interactions [7]. Although emotional expressions account for much of this communication, static features in the face may also provide information such as formidability and aggressiveness; such static cues have been described in other species (e.g., black facial pattern of paper wasps, *Polistes dominulus* [8]). There is abundant evidence that humans form snap judgements of dominance and threat (e.g., aggressiveness, strength, fighting ability) [9]. Additionally, there is evidence that such judgements are accurate: people who were judged as more powerful reported being higher in assertiveness, social potency, aggressiveness, and power [10]; those who were judged as stronger and better at fighting were physically stronger and reported fighting more frequently [9]; and criminals who were judged as more violent were more likely to have been incarcerated for violent than for non-violent crimes than were criminals who were judged as less violent [11].

**Fig 1:**
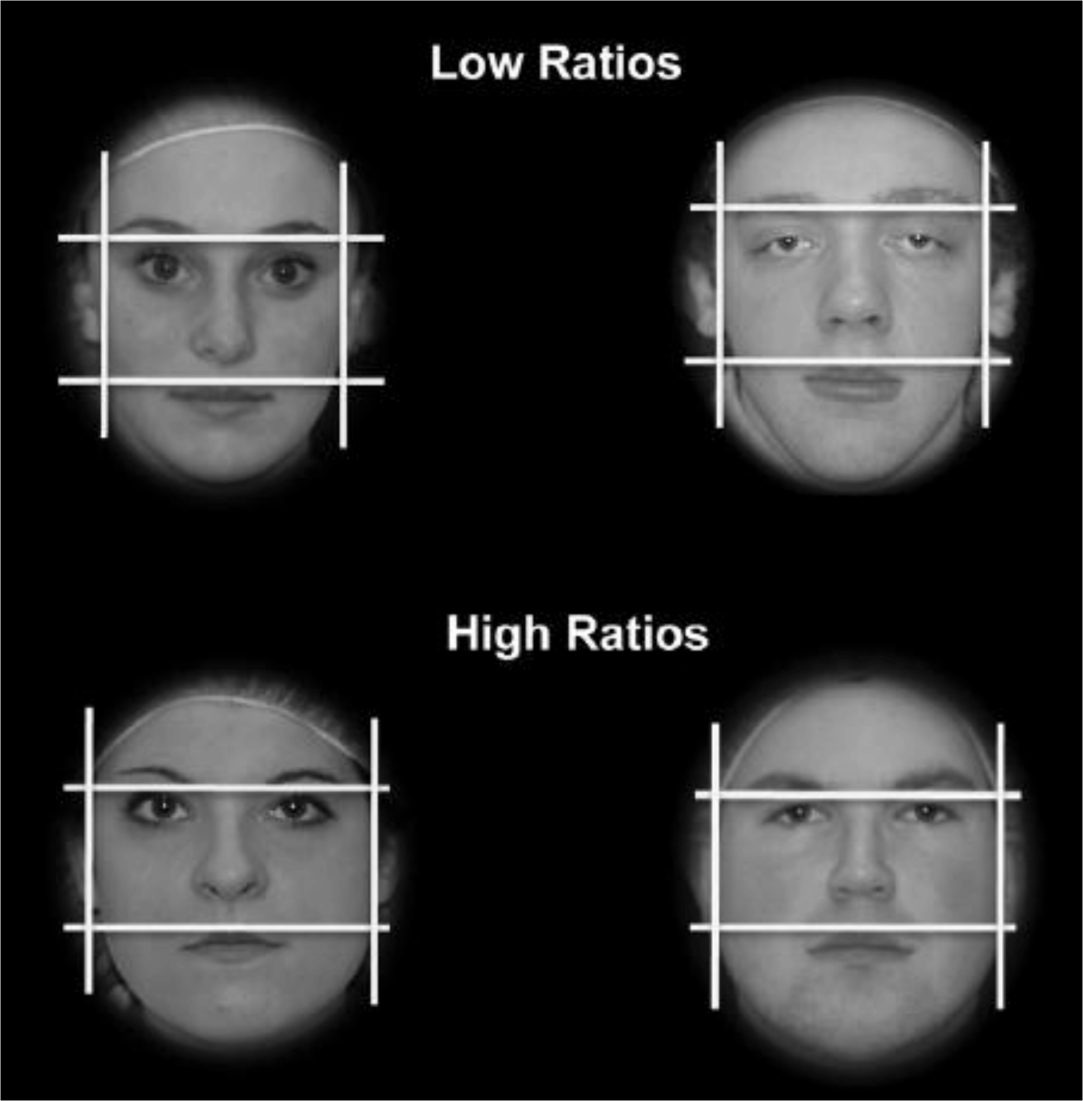
Examples of measurement of the FWHR in faces with relatively low and high FWHRs.

The facial width-to-height ratio (FWHR; the width of the face divided by the height of the upper face) may be an important static cue of threat; it is perceived rapidly [12], it is conspicuous even in bearded men [13], and it predicted men’s aggressive behaviour both in and outside of the laboratory [14,15]. Observers’ estimates of aggression, dominance, and formidability are reliably correlated with the FWHR (e.g., [16,17]). Further, the FWHR is positively associated with dominance in non-human primates [18], and humans can accurately assess this trait in non-human primates [19,20], suggesting that the FWHR, and sensitivity to it, may be part of an evolved cuing system in human and non-human primates. Nevertheless, the reliability of these relationships (e.g., [21,22]) and the report of a larger FWHR in men than in women (e.g., [23,24]) have been questioned (see Fig 1 for examples of faces with relatively low and with relatively high FWHRs).

### The current meta-analytic review

The abundance of research on the FWHR since its first report in humans [25] permits an assessment of the reliability and magnitude of these relationships. Although a meta-analysis on the FWHR was recently published [26], the scope of that analysis was limited to characterizing the relationship between the FWHR and aggression among men only. Here, we systematically review a greater body of FWHR research and we use meta-analyses to investigate whether this metric: (1) is sexually dimorphic; (2) cues judgements of threat and dominance across several domains; and (3) is an accurate index of these characteristics and behaviours in both men and women. In so doing, we provide a more definitive test of the hypothesis that the FWHR is part of an evolved cueing system of intra-sexual threat, dominance, and aggressiveness in men, akin to those in other species (e.g., [8]).

## Materials and Methods

We identified all peer-reviewed and published or in-press manuscripts written in English that contained effect sizes related to the FWHR by using the search term “facial width-to-height ratio” in Google Scholar and by searching for citations of Weston, Friday, & Liò [25], the first article published on the FWHR (our search ended December 31^st^, 2014). We also included effect sizes from four separate manuscripts that were submitted for review by authors of the current manuscript (Denson, unpublished manuscript; Yang, Chao, Fabiansson, & Denson, unpublished manuscript; two of which have been accepted since [13,27]) and one manuscript of the authors that included the term “facial width-to-height ratio” [28] but was not detected by Google Scholar. This strategy identified 63 peer-reviewed manuscripts. Effect sizes from seven of these manuscripts were not used in any of the meta-analyses, however, because they either did not conduct analyses that were relevant to our research questions [29–31], involved non-human primates [18,32,33], or used faces intentionally posed in non-neutral expressions [34]. Therefore, analyses were conducted on effect sizes extracted from a total of 56 manuscripts.

We used an effect size determination program [35] and formulas provided in Bonett [36] and Tabachnick and Fidell [37] to convert effect sizes to either a Pearson product moment correlation (*r*) or to a standardized mean difference (*d*). When estimating effect sizes from studies using multilevel modelling or binary logistic regression, we converted the *χ* ^2^ values from the individual predictors to *r* or *d* values using Wilson’s [35] effect size determination program, or we computed a *t* value by dividing the coefficient by the standard error of the coefficient (as in [38]), and converted this value to an *r* or *d* value using Wilson’s program [35]. When standardized coefficients (*ß* weights) were provided instead of *r* values, we used *ß* weights as direct estimates of *r* values given their equivalence when a variable is entered as the sole predictor in a regression, and their strong correlation when the variable of interest is entered along with several other simultaneous predictors in a regression [39]. When ^2^ values were provided, we used the square root of these values as an estimate of the *r* effect size. Three of the authors coded all effect sizes; discrepancies were resolved through discussion. Additional detail regarding data extraction and effect size conversions are in the S1 File.

For meta-analyses involving the *d* effect size values, the *d*s were adjusted to correct for small sample size bias [*d*(1-(3/(4*N* – 9)))] [40] and were weighted by the inverse variance (1/*se* ^2^) before calculation of the mean weighted effect size. Therefore, all *d* (for individual effect sizes) and $\bar{d}$ (for mean weighted effect sizes) values are presented in the adjusted, unbiased form in tables, figures, and text unless otherwise stated. As recommended [41], for meta-analyses involving the *r* effect size values, the *r*s were transformed to Fisher z correlations and weighted by the inverse variance (*N*– 3) before calculating the mean weighted effect size. For ease of interpretation, however, these Fisher z estimates were then transformed back into their standard *r* (for individual effect sizes) or their $\bar{r}$ (for mean weighted effect sizes) form when presented in tables, figures, and text.

The data were analyzed using SPSS macros with random-effects models [35]. The macro “MEANES” was used to determine the mean weighted effect sizes; the macro “METAF” was used to test individual moderators with two levels. When an individual moderator with two levels was significant, the file was split by the moderating variable and the macro “MEANES” was used to determine the mean weighted effect size within each level or subgroup. The macro “METAREG” was used to test moderators with continuous values, or was used to test multiple moderators (with discrete levels, or with continuous values) simultaneously (e.g., to test the effect of one moderator, statistically controlling for the other moderators). Although we present results separately for each subgroup when a moderator with two levels was tested independently and found to be significant, we only provide *B* weights when the moderator had continuous rather than ordinal values or when it was tested simultaneously with other moderators. The *B* weights can be used to determine the extent to which the mean weighted effect size changes with each unit change of the moderator variable (controlling statistically for any other moderators that may be included in the model). Therefore, if a moderator had a *B* weight of .20, the strength of the mean weighted effect size increases by .20 with a one unit increase in the moderator. Similarly, if the moderator involved two levels, it would suggest that the relationship within one level differed .20 from the relationship within the other level. All moderators were tested separately (without other moderators in the model) unless otherwise specified.

Our meta-analysis on the relationship between the FWHR and threat differs from that of Haselhuhn and colleagues [26] in that we included a broader array of behaviours (e.g., prejudice, financial misreporting) related to threat, and also investigated the association in both men and women. Although there are discrepancies in the definition of “threat” in the literature, we use the term according to its definition in the Merriam-Webster dictionary (http://www.merriam-webster.com/dictionary/threat): “someone or something that could cause trouble, harm, etc.” Because this definition is broad, it captures many related yet distinct behaviours (e.g., aggression, prejudice, deception). We therefore conducted moderator analyses to examine whether the association between the FWHR and threat differs in strength depending on the type of threat; we distinguished between the most commonly investigated type of threat, aggressive behaviour, and other selfish and pejorative behaviours.

**Fig 2:**
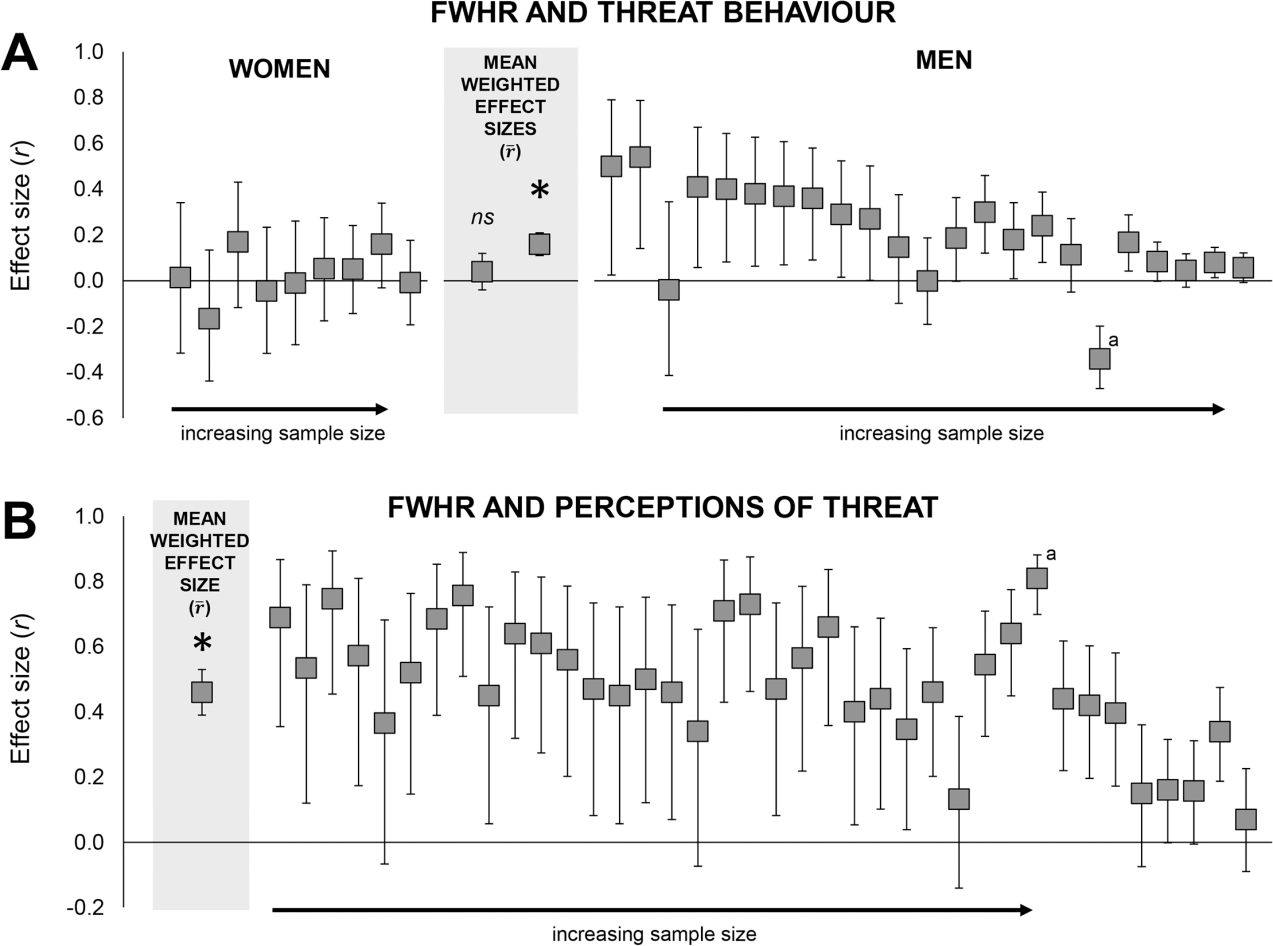
Effect sizes (*r*s) included in the meta-analysis on the relationships between the FWHR and threat behaviour (Panel A) and between the FWHR and perceptions of threat (Panel B). The mean weighted effect sizes **($\bar{r}s$)** are highlighted in grey, with men and women separated for Panel A and combined for Panel B. *p < .0001. ^a^effect size was removed from the final analysis.

Our analysis also differs from that of Haselhuhn and colleagues [26] in that we estimated the means and standard deviations from Fig 2B of Gómez-Valdés and colleagues [21] rather than assume the relationship between the FWHR and threat behaviour was *r* = .00. Compared to the analysis of Haselhuhn and colleagues [26], which included 4141 men from 18 samples, our analysis in men included 4573 participants (and 30 male dyads) from 23 samples. Again, the samples included in our analysis were derived from studies involving a broader array of behaviours related to threat than those included in Haselhuhn and colleagues’ [26] analysis. We excluded a study that investigated death by contact violence [42] because this study examined aggression towards, rather than aggression perpetrated by, the individual; this study, however, was in the meta-analysis of Haselhuhn and colleagues [26]. We also used a random-effects model to analyse all data rather than use a fixed-effects model, which was used by Haselhuhn and colleagues [26].

When deciding which effect sizes to extract for examining the relationship between the FWHR and dominance, we referred to the definition of dominance in the Merriam-Webster dictionary, one’s relative position within a social hierarchy (http://www.merriam-webster.com/dictionary/dominance), as well as questionnaire measures of dominance and prestige (e.g., “I do NOT have a forceful or dominant personality” (reversed); “I try to control others rather than permit them to control me”; “I often try to get my own way regardless of what others may want”; “Others always expect me to be successful;” [43]; “Have a strong need for power”; [44]). The analysis included effect sizes related to self-perceived, other-perceived, or objectively determined prestige, forcefulness, inflexibility, competitiveness, military rank, sense of power, and achievement drive.

For moderator analyses, we extracted information related to the nationality and mean age of the samples and, if a study involved observers’ perceptions, the number of observers and the mean age and nationality of the faces used as stimuli. We also extracted information on the measurement of the FWHR (2D photos, 3D scans, etc; see S1 File for additional notes regarding moderators) for the analysis of sex differences in the size of the FWHR. Although we provide funnel plots for each analysis involving 10 or more effect sizes [45] (see S1 Fig), we caution that asymmetry in the plots may arise for a number of reasons other than, or in addition to, publication bias (e.g., true heterogeneity in the effect size, poor methodological design of smaller studies, chance; reviewed in [46]). When there were significant moderators of effect sizes and the moderators were discrete variables (rather than continuous), we provide the funnel plots within each subgroup (unless the subgroup involved a small number of studies, *k* < 10). We also provide a fail-safe *n* [41,47] for each significant effect (*p* ≤ .05), indicating the number of additional studies with null effects that would have to be added to the analysis to make the magnitude of the mean weighted effect size trivial, $\bar{r}$ = .01 or $\bar{d}$ = 0.01. We visually inspected the funnel plots for any potential outliers. For each meta-analysis, we report the number of samples that were included (*k*). The PRISMA Checklist [48] is provided in S3 File.

## Results

Table 1 provides a summary of the results.

**Table 1:** Summary of the final results of the meta-analyses conducted in the current manuscript.

Analysis	*k*	Mean Weighted Effect Sizes	95% CI (low)	95% CI (high)
Sex differences in the FWHR	32	$\bar{d}$ = **0.11**	0.03	0.20
FWHR and Perceptions of Masculinity	12	$\bar{r}$ = **.30**	.18	.42
Stimuli sets of male faces	10	$\bar{r}$ = **.35**	.23	.47
Stimuli sets of female faces	2	$\bar{r}$ = -.01	-.26	.25
FWHR and Threat and Dominance Behaviour				
Threat Behaviour	31	$\bar{r}$ = **.13**	.09	.17
Within men	22	$\bar{r}$ = **.16**	.11	.21
Within women	9	$\bar{r}$ = .04	-.04	.12
Within Samples from North America	9	$\bar{r}$ = **.25**	.17	.33
Within Samples from Other areas	13	$\bar{r}$ = **.12**	.07	.17
Dominance Behaviour	16	$\bar{r}$ = **.12**	.05	.18
Success in Business-Related Outcomes	5	$\bar{r}$ = **.32**	.12	.50
Sports Performance	4	$\bar{r}$ = **.15**	.08	.22
FWHR and Perceptions of Threat and Dominance				
Perceptions of Threat				
Studies using a correlational design and/or a continua of faces with un-manipulated FWHRs	37	$\bar{r}$ = **.46**	.39	.53
Judgements were more strongly linked to the FWHR when faces were of younger than older individuals (*k* = 26, *B* = .40, *p* < .0001)				
Judgements of aggression were more strongly linked to the FWHR than were other types of judgements of threat (*k* = 26, *B* = .15, *p* = .01)				
Studies using manipulated FWHRs	7	$\bar{d}$ = **0.41**	0.29	0.53
Perceptions of Dominance	7	$\bar{r}$ = **.20**	.06	.34
Stimuli sets of male faces only	4	$\bar{r}$ = **.30**	.19	.40
Stimulus sets of or including female faces	3	$\bar{r}$ = .06	-.19	.31
FWHR and Perceptions of Attractiveness	14	$\bar{r}$ = **-.26**	-.40	-.10
The negative relationship between judgements of attractiveness and the FWHR was stronger among samples with a greater than a lesser proportion of female observers (*k* = 14, *B* = .008, *p* = .01)				
FWHR and BMI	22	$\bar{r}$ = **.31**	.26	.36

Bolded effect sizes are significant and have confidence intervals that do not overlap zero (*p* < .05). *k* = number of samples included in the analysis. $\bar{d}$ = standardized mean difference, adjusted for small sample size bias. $\bar{r}$ = untransformed effect size coefficient (Pearson product moment correlation). CI = confidence interval.

### Are men’s FWHRs larger than women’s?

Studies were included in the analysis if they reported statistics comparing the FWHR of men and women, or descriptive statistics regarding the size of the FWHR (means, *SD*) for the sexes separately. With these inclusion criteria, 19 of the 56 manuscripts were included in the analysis. Effect sizes were extracted from 32 samples involving 6113 men and 4740 women (Table A in S2 File) (*M*_age_ = 25.19 years; range: 18.98–83). Men had slightly larger FWHRs than did women (*k* = 32, $\bar{d}$ = 0.11, *95% CI* = 0.03 to 0.20, *p* = .009; *Q*_31_ = 110.49, *p* < .0001; fail-safe *n* = 320), even after removing the largest outlying effect size (*k* = 31, $\bar{d}$ = 0.08, *95% CI* = 0.003 to 0.16, *p* = .04; *Q*_30_ = 80.32, *p* < .0001; fail-safe *n* = 310). Neither age, measurement type (2D photographs vs other), nor nationality (North American vs other) moderated the effect (*p*s > .21).

### Are larger FWHRs perceived as more masculine than are smaller FWHRs?

Studies were included in the analysis if they reported statistics examining the association between the FWHR and judgements of masculinity or of femininity (femininity correlations were reversed for the analysis). With these inclusion criteria, six of the 56 manuscripts were included in the analysis using correlational design and/or a continuum of faces with un-manipulated FWHRs.

### Studies using a correlational design and/or a continuum of faces with un-manipulated FWHRs

Effect sizes were extracted from 12 samples (Table B in S2 File), which involved a total of 139 male observers from 9 of the samples and 200 female observers from 11 of the samples (*M*_age_ = 25.70). The stimuli included 425 male faces from 10 of the samples and 62 female faces from two of the samples (*M*_age_ = 19.42). The mean weighted relationship between the FWHR and perceptions of masculinity was positive and significant (*k* = 12, $\bar{r}$ = .30, *95% CI* = .18 to .42, *p <* .0001; *Q*_11_ = 19.53, *p* = .05; fail-safe *n* = 348). Sex of the stimuli moderated the effect (*k* = 12, *Q*_1_ = 4.62, *p* = .03), with stronger effects in male (*k* = 10, $\bar{r}$ = .35, *95% CI* = .23 to .47, *p <* .0001; *Q*_9_ = 14.63, *p* = .10; fail-safe *n* = 340) than in female faces (*k* = 2, $\bar{r}$ = -.01, *95% CI* = -.26 to .25, *p* = .97; *Q*_1_ = 0.11, *p* = .74). Although the relationship between the FWHR and perceptions of masculinity/femininity differed for male and female faces, there were only two samples from which the estimate for female faces was derived. Further, there were only 31 unique female facial identities used in the analysis. Therefore, future studies would benefit from examining this potential moderating factor using a larger set of unique female faces as stimuli.

Within the samples using male faces as stimuli, neither the number of observers, age of observers, nor age of stimuli moderated the effect (*p*s > .21). The percentage of male observers was a significant moderator (*k* = 10, *B* = -.004, *p* = .003), but the effect was driven by one effect size [49]. After its removal, the moderator was not significant (*k* = 9, *B* = .000, *p* = .91). Among studies using male faces as stimuli, the most frequently used stimuli set (Carré and colleagues [16]) did not produce stronger effect sizes than did studies involving other stimuli sets (*p* = .59).

### Studies using faces with manipulated FWHRs

No studies to date examined perceptions of masculinity between two versions of a face manipulated to have smaller versus larger FWHRs.

### Does the FWHR predict threatening and dominant behaviour?

#### Threat

Nineteen of the 56 manuscripts met the inclusion criteria for the analysis of the association between the FWHR and threat behaviour (selfish, pejorative, and as aggressive behaviour). Effect sizes were extracted from 32 samples (Table C in S2 File). There was a total of 4573 men (and 30 male dyads) from 23 of the samples and 634 women from 9 of the samples (*M*_*age*_ = 21.77 years; range: 18.98–28). The FWHR predicted threat behaviour (*k* = 32, $\bar{r}$ = .12, *95% CI* = .07 to .17, *p <* .0001; fail-safe *n* = 352) despite the presence of one apparent outlier [21], which was the only effect size with confidence intervals that did not overlap with those of the mean weighted effect size (*r* = -.34, *95% CI* = -.47 to-.20) (Fig 2). Note that the outlying effect size was computed by comparing the FWHRs of the general population to the weighted mean of three criminal groups (persecuted by homicide, by robbery, by other minor faults, see Fig 2B of [21] and footnote in Table C of S2 File). Nevertheless, the FWHR may be related to socioeconomic success (given its links with performance in economic negotiations and business; see analysis below), which is known to predict criminality (e.g., [50]), thus representing a potential suppression effect. In support of this possible suppression effect, when we minimize the influence of socioeconomic status by making comparisons within the criminal group [comparing the group persecuted by homicide (*n* = 58, mean = 1.838, *SD* = 0.118) to the weighted mean of those persecuted by robbery and other minor faults (total *n* = 49, weighted mean = 1.803, pooled *SD* = 0.111)] the effect size becomes positive (*r* = .15) and more consistent with the mean weighted effect size reported for men.

Excluding this outlying effect size increased the mean weighted effect size, tightened the confidence interval (*k* = 31, $\bar{r}$ = .13, *95% CI* = .09 to .17, *p <* .0001; fail-safe *n* = 372), and reduced the heterogeneity (outlier included: *Q*_31_ = 83.60, *p <* .0001; outlier excluded: *Q*_30_ = 50.96, *p* = .01). Because this analysis also involved effect sizes from studies that investigated fighting ability [17,51], which involves a combination of aggressiveness and athletic ability, we re-ran the analysis without these studies included. The mean weighted effect size was unchanged although the confidence intervals became slightly wider (*k* = 29, $\bar{r}$ = .13, *95% CI* = .09 to .18, *p* < .0001; fail-safe *n* = 360). This analysis also involved some effect sizes that may have come from overlapping samples in different manuscripts (UFC fight performance [17,51]; penalty minutes of players from the National Hockey League [14,15,52]. When we included only the effects size from the largest sample of the overlapping studies involving UFC fighters [17] and of the overlapping studies involving hockey players [15], the mean weighted effect size was unchanged (*k* = 28, $\bar{r}$ = .13, *95% CI* = .08 to .18, *p* < .0001; fail-safe *n* = 348).

Sex interacted with the FWHR (*k* = 31, *Q*_1_ = 5.37, *p* = .02); the relationship was significant only in men (*k* = 22, $\bar{r}$ = .16, *95% CI* = .11 to .21, *p <* .0001; *Q*_21_ = 43.15, *p* = .003; fail-safe *n* = 330) (women: *k* = 9, $\bar{r}$ = .04, *95% CI* = -.04 to .12, *p* = .34; *Q*_8_ = 4.91, *p* = .77; Fig 2). Note that the interaction involving sex was marginally significant when the outlying effect size from Gómez-Valdés and colleagues [21] was included in the analysis (*k* = 32, *Q*_1_ = 3. 53, *p* = .06). The type of threat (aggressive vs selfish and pejorative) was not a moderator (*k* = 22, *Q*_1_ = 2.38, *p* = .12), with the FWHR predicting both aggressive (*k* = 12, $\;\bar{r}$ = .13, *95% CI* = .07 to .18, *p <* .0001; *Q*_11_ = 19.72, *p* = .05; fail-safe *n* = 144) and selfish/pejorative behaviour (*k* = 10, $\bar{r}$ = .25, *95% CI* = .14 to .35, *p <* .0001; *Q*_9_ = 21.78, *p* = .01; fail-safe *n* = 240) in men. The type of measure (self-report vs behavioural) did not moderate the relationship (*k* = 22, *Q*_1_ = 0.18, *p* = .67) (behavioural measures: *k* = 17, $\bar{r}$ = .17, *95% CI* = .11 to .22, *p* < .0001; *Q*_16_ = 36.96, *p* = .002; fail-safe *n* = 272; self-report measures: *k* = 5, $\bar{r}$ = .14, *95% CI* = -.01 to .29, *p* = .07; *Q*_4_ = 6.13, *p* = .19). Nationality (North American vs other) moderated the relationship (*k* = 22, *Q*_1_ = 6.65, *p* = .01); the FWHR shared stronger relationships with threat behaviour when effect sizes were derived from North American (*k* = 9, $\bar{r}$ = .25, *95% CI* = .17 to .33, *p* < .0001; *Q*_8_ = 7.85, *p* = .45; fail-safe *n* = 216) than from other samples (*k* = 13, $\bar{r}$ = .12, *95% CI* = .07 to .17, *p* < .0001; *Q*_12_ = 23.05, *p* = .03; fail-safe *n* = 143), although the mean weighted effect was significant irrespective of the nationality. Age did not moderate the relationship between the FWHR and threat behaviour (*p* = .42).

#### Dominance

Ten of the 56 manuscripts met the inclusion criteria for the analysis. Effect sizes were derived from 17 samples (Table D of S2 File), with a total of 1426 men (and 30 male dyads) from 11 of the samples and 287 women from 6 of the samples (*M*_age_ = 22.04 years; range: 18.98–33.61). All studies involved subjective measures of dominance (either self-report or, for a study involving previous presidents [53], inferred dominance) except one [54] that involved the relationship between the FWHR and military rank of Finnish soldiers at the start of World War II. The relationship between the FWHR and dominance was positive and significant (*k* = 17, $\bar{r}$ = .10, *95% CI* = .002 to .20, *p* = .05; *Q*_16_ = 45.16, *p* = .0001; fail-safe *n* = 153). When only studies that involved self-reported or inferred measures of dominance were included, the confidence interval was tighter and the distribution of effect sizes was no longer heterogeneous (*k* = 16, $\bar{r}$ = .12, *95% CI* = .05 to .18, *p* = .0005; *Q*_15_ = 14.68, *p* = .47; fail-safe *n* = 176), likely because the study of Finnish soldiers [54] produced the only effect size with confidence intervals that did not overlap those of the mean weighted effect size. Sex did not moderate the relationship (*k* = 16, *Q*_1_ = 0.20, *p* = .65) (men: *k* = 10, $\bar{r}$ = .14, *95% CI* = .04 to .24, *p* = .008; *Q*_9_ = 13.59, *p* = .14; fail-safe *n* = 130) (women: *k* = 6, $\bar{r}$ = .09, *95% CI* = -.03 to .21, *p* = .12; *Q*_5_ = 0.91, *p* = .97). Neither nationality (North American vs other) nor age moderated the relationship between the FWHR and dominance (*p*s > .29).

We included the studies of business-related outcomes (any effect sizes related to negotiation abilities, business position) and sports performance (any effect sizes related to wins and indices of successful performance in sports, e.g., assists, goals) as additional indices of dominance. With the inclusion criteria, 4 of the 56 manuscripts were included in the analysis on business-related outcomes. The analysis included effect sizes from 6 samples (Table E of S2 File) involving a total of 241 men (and 87 male dyads and 86 male groups) (*M*_age_ = 27.14). The FWHR predicted success in business, marginally (*k* = 6, $\bar{r}$ = .22, *95% CI* = -.04 to .46, *p* = .09; *Q*_5_ = 29.23, *p <* .0001). The association was negative in only one study (Study 3 of [55]), which was similar to other studies included in the analysis in that it examined the ability to negotiate, but differed from other studies in that it assessed the ability to negotiate legitimately (within the rules of the bargaining exercise). This effect size may have been opposite to the other effect sizes because it represents a measure of bargaining within the rules of the bargaining game. Nevertheless, as the analyses above suggest, men with larger FWHRs are more antisocial than those with smaller FWHRs and this effect may thus be driven by an increased likelihood of “cheating” in the task to achieve the goal. When this effect size was excluded, the mean weighted effect became significant and had narrower confidence intervals (*k* = 5, $\bar{r}$ = .32, *95% CI* = .12 to .50, *p* = .002; *Q*_4_ = 13.61, *p* = .009; fail-safe *n* = 155). Neither nationality (North American vs other) nor age moderated the relationship (*p*s > .60).

Four of the 56 manuscripts met the inclusion criteria for the analysis of sports performance. The analysis included effect sizes from 4 samples (Table F of S2 File) involving a total of 1401 men (*M*_age_ = 29.34). The FWHR predicted sports performance (*k* = 4, $\bar{r}$ = .10, *95% CI* = .005 to .19, *p* = .04; *Q*_3_ = 6.57, *p* = .09; fail-safe *n* = 36). One of the samples [56] included a measure of performance in soccer players (the average of the associations between the FWHR and assists and between the FWHR and goals). The authors performed analyses controlling for player position (defender, midfielder, forward) and also within each player position. Because forwards have more opportunities to score goals and make more assists than do midfielders and defenders [56], we also examined the mean weighted association between the FWHR and sports performance when this subsample of forwards (*n* = 211) was used instead of the entire sample. The mean weighted effect size from this analysis was stronger, and the heterogeneity was reduced (*k* = 4, $\bar{r}$ = .15, *95% CI* = .08 to .22, *p* = .0001; *Q*_3_ = 0.35, *p* = .95; fail-safe *n* = 56).

### Are perceptions of threat and dominance associated with the FWHR?

#### Studies of threat using a correlational design and/or a continuum of faces with un-manipulated FWHRs

For the analysis of perceived threat, we included any studies that reported statistical analyses on the relationship between the FWHR and threat-related judgements (see definition of threat in methods). These judgements included those of aggressiveness, untrustworthiness, formidability (strength, toughness, fighting ability, physical power), and prejudice. With these inclusion criteria, 18 of the 56 manuscripts were included in the analysis of studies that used a correlational design and/or a continuum of faces with un-manipulated FWHRs. Effect sizes were extracted from 38 samples (Table G of S2 File) involving a total of 779 male observers from 36 of the samples and 1313 female observers from all 38 of the samples (*M*_age_ = 21.57) (see Fig 2). The stimuli included 1679 male faces from 36 of the samples and 72 female faces from three of the samples (*M*_age_ = 22.64). The FWHR predicted perceptions of threat (*k* = 38, $\bar{r}$ = .48, *95% CI* = .41 to .55, *p <* .0001; *Q*_37_ = 125.43, *p <* .0001; fail-safe *n* = 1786). When we removed the largest effect size (*r* = .81, Study 3 of [57]), the strength of the association decreased slightly (*k* = 37, $\bar{r}$ = .46, *95% CI* = .39 to .53, *p <* .0001; *Q*_36_ = 96.71, *p <* .0001; fail-safe *n* = 1739).

Because a cluster of the variables we investigated as moderators were correlated with one another (all *r*s > .28, *p*s < .10), we entered them as simultaneous moderators [number of observers, nationality of the observers (North American vs other), nationality of the faces used as stimuli (North American vs other), age of the faces used as stimuli (younger than 25 vs older than 25). Only the age of the stimuli emerged as a significant moderator (*k* = 25, *B* = -.28, *p* = .01) (all other *p*s > .34), with perceptions of threat sharing stronger links with the FWHR of younger compared with older individuals. The percentage of male observers, the age of observers, whether the stimuli included female faces, and the type of the judgement (judgements of only aggression vs other), were not significant moderators (all *p* > .10). When these variables were entered as simultaneous moderators along with the age of the stimuli faces, the only significant moderators were age of the stimuli faces (*k* = 26, *B* = -.40, *p* < .0001) and judgement type (*k* = 26, *B* = .15, *p* = .01); the FWHR predicted perceptions of threat more strongly in younger than in older faces, and when participants’ judgements were of aggression on its own compared with when other threat judgements were involved.

We also examined whether there were any differences in the strength of the association between the FWHR and judgements of threat when effect sizes were obtained from studies using the most common stimuli set (24 male faces from Carré and colleagues [16]) compared to other stimuli sets. Studies using the stimuli set from Carré and colleagues [16] (*k* = 11, $\bar{r}$ = .61, *95% CI* = .52 to .68, *p <* .0001; *Q*_10_ = 7.57, *p =* .67; fail-safe *n* = 660) produced stronger effect sizes (*k* = 37, *Q*_1_ = 9.02, *p* = .003) than did studies using other stimuli sets (*k* = 26, $\bar{r}$ = .40, *95% CI* = .32 to .48, *p <* .0001; *Q*_25_ = 64.82, *p <* .0001; fail-safe *n* = 1014). Because the Carré and colleagues [16] stimuli set involved younger faces, was more often used to assess perceptions of aggression, and was more often rated by North American observers, compared with other stimuli sets (all *r*s > .30), we examined whether these three variables explained its stronger associations. When these three moderators (age of faces used as stimuli, nationality of the observers, judgement type) were entered simultaneously with the type of stimuli set (Carré and colleagues vs other), only the age of the faces emerged as a significant moderator (*k* = 25, *B* = -.32, *p* = .0007; all other *p*s >.05), suggesting that these variables may, in part, explain the stronger associations obtained when studies used the Carré and colleagues stimuli set compared to other stimuli sets.

#### Studies of threat using faces with manipulated FWHRs

Six of the 56 manuscripts met the inclusion criteria for the analysis of studies involving faces with manipulated FWHRs. Effect sizes were extracted from 11 samples (Table H of S2 File) involving a total of 467 male observers from all 11 samples and 9135 female observers from all 11 of the samples as well (*M*_age_ = 25.78). Faces with larger FWHRs were rated as more threatening than those with smaller FWHRs, but the difference missed statistical significance (*k* = 11, $\bar{d}$ = 0.42, *95% CI* = -0.02 to 0.86, *p* = .06; *Q*_10_ = 155.65, *p* < .0001). The heterogeneity was driven by one outlying effect size (Study 1 of [57]), which was removed from subsequent analyses, and three effect sizes that were in a direction opposite to that of the other seven effect sizes. The three negative effect sizes were derived from studies using small stimulus sets (one manipulated stimulus face, [58]; two male and two female manipulated stimulus faces, [59]), which may have obscured the relationship between the FWHR and perceptions of threat. Consistent with this possibility, the number of base stimulus images or composites moderated the strength of the effect size (*k* = 10, *B* = .09, *p* < .0001), such that studies that utilized more base stimulus images or composites produced larger effect sizes than those that used fewer. In addition to the size of the stimulus set, it is also possible that two of the three effect sizes were in a direction opposite to that of the rest of the studies because they came from a study that involved the use of avatars that were caricatured rather than realistic in appearance (see Fig 1 of [59]). Further, for the other negative effect size, the manipulation of the FWHR may have incidentally exaggerated the lower jaw and increased perceptions of adiposity, which may have influenced the judgements (see Fig 3 of [58]).

After excluding the studies that used only one or two base images, faces manipulated to have larger FWHRs were perceived as significantly more threatening than those manipulated to have smaller FWHRs, and heterogeneity was reduced (*k* = 7, $\bar{d}$ = 0.41, *95% CI* = 0.29 to 0.53, *p* < .0001; *Q*_6_ = 5.12, *p* = .53; fail-safe *n* = 287). In this smaller sample of effect sizes (*k* = 7), neither threat type (aggression vs other), sex of stimuli, percentage of male observers, nationality of the stimuli (North America vs other), nor nationality of the observers (UK vs other) (*p*s > .12) moderated the effect.

#### Studies of dominance using a correlational design and/or a continuum of faces with un-manipulated FWHRs

For the analysis of perceived dominance, we included any studies that reported statistical analyses on the relationship between the FWHR and dominance-related judgements (see definition of dominance in methods). Seven of the 56 manuscripts met the inclusion criteria for the analysis involving studies that used a correlational design and/or a continuum of faces with un-manipulated FWHRs. Effect sizes were extracted from 8 samples (Table I of S2 File) involving a total of 107 male observers from all eight of the samples and 153 female observers from all eight of the samples (*M*_age_ = 24.88). The stimuli included 461 male faces from seven of the samples and 202 female faces from three of the samples (*M*_age_ = 28.89). The FWHR predicted perceptions of dominance (*k* = 8, $\bar{r}$ = .29, *95% CI* = .10 to .47, *p* = .004; *Q*_7_ = 45.08, *p <* .0001; fail-safe *n* = 224). Nevertheless, one effect size seemed to be driving the effect (Study 3 of [57]); after excluding the study, the effect size decreased in magnitude but was still positive and significant (*k* = 7, $\bar{r}$ = .20, *95% CI* = .06 to .34, *p* = .007; *Q*_6_ = 18.70, *p* = .005; fail-safe *n* = 196). The number of observers, percentage of male observers, age of observers, nationality of observers (North America vs other), age of stimulus faces, and nationality of stimulus faces (North America vs other) did not moderate the relationship between the FWHR and perceived dominance (*p*s > .19). The relationship between the FWHR and perception of dominance, however, was marginally stronger (*k* = 7, *Q*_1_ = 3.62, *p* = .06) when stimuli sets were exclusively male faces (*k* = 4, $\bar{r}$ = .30, *95% CI* = .19 to .40, *p <* .0001; *Q*_3_ = 2.53, *p* = .47, fail-safe *n* = 140) than when they were not (*k* = 3, $\bar{r}$ = .06, *95% CI* = -.19 to .31, *p* = .64; *Q*_2_ = 9.07, *p* = .01).

#### Studies of dominance using faces with manipulated FWHRs

Only one study that manipulated the FWHR and investigated changes in perceptions of dominance fit the inclusion criteria [58]. The study manipulated a single male face to have a larger versus a smaller FWHR and reported significantly higher ratings of dominance for the version of the face with the larger than the smaller FWHR (*unadjusted d* = 0.61, 52 observers). This effect was not included in any of the meta-analyses.

### Is the FWHR associated with perceived attractiveness?

#### Studies using a correlational design and/or a continuum of faces with un-manipulated FWHRs

For the analysis on perceived attractiveness, we included any studies that reported statistical analyses on the relationship between the FWHR and attractiveness-related judgements (attractiveness, short-term or long-term desirability as a romantic partner). Nine of the 56 manuscripts met the inclusion criteria for the analysis involving studies that used a correlational design and/or a continuum of faces with un-manipulated FWHRs. Effect sizes were extracted from 14 samples (Table J of S2 File) involving a total of 106 male observers from 10 of the samples and 229 female observers from all 14 of the samples (*M*_age_ = 24.81). The stimuli included 659 male faces from 12 of the samples and 62 female faces from two of the samples (*M*_age_ = 22.84). The relationship between the FWHR and perceptions of attractiveness was negative and significant (*k* = 14, $\bar{r}$ = -.26, *95% CI* = -.40 to-.10, *p* = .001; *Q*_13_ = 50.69, *p* < .0001, fail-safe *n* = 350). Neither the number of observers, age of observers, age of the stimuli, sex of the stimuli, the nationality of the stimuli (North American vs other), nor the nationality of the observers (North American vs other) moderated the effect (all *p*s > .15). The percentage of male observers, however, did moderate the effect (*k* = 14, *B* = .008, *p* = .01); the negative relationship between the FWHR and judgements of attractiveness was stronger when the sample had a greater proportion of women than men, suggesting that faces with larger FWHRs may be especially unattractive to female observers. The strength of the mean weighted effect size did not differ between studies using the most frequently used stimuli set, that of Carré and colleagues [16], and other stimuli sets (*p* = .65).

#### Studies using faces with manipulated FWHRs

Only one study that manipulated the FWHR and investigated changes in perceptions of attractiveness met the inclusion criteria [58]. The study manipulated a single male face to have a larger versus a smaller FWHR and reported no significant differences between the ratings of attractiveness for the two versions of the faces (*unadjusted d* = 0.06, 55 observers). This effect was not included in any of the meta-analyses.

### Is the FWHR associated with Body Mass Index (BMI)?

For the analysis on the FWHR and BMI, we only included studies that reported statistical analyses on the relationship between the FWHR and BMI; we did not include associations with other indices of size or adiposity. BMI may mediate the relationship between the FWHR and behaviour. Nine of the 56 manuscripts met the inclusion criteria for the analysis. Effect sizes were extracted from 22 samples (Table K of S2 File): There was a total of 1479 men from 16 of the samples and 1009 women from 11 of the samples (some samples involved both male and female participants and did not report results separately for men and women) (*M*_age_ = 25.19 years; range: 19.6–83). The mean weighted relationship between the FWHR and BMI was positive and significant (*k* = 22, $\bar{r}$ = .31, *95% CI* = .26 to .36, *p <* .0001; *Q*_21_ = 34.62, *p* = .03, fail-safe *n* = 660). Neither nationality (UK vs other), sex, nor age moderated the relationship between the FWHR and BMI (*p*s > .28).

### Examination of funnel plots

See S1 Fig for funnel plots. The funnel plots indicate that the distribution of effect sizes for most of the meta-analyses were symmetrical, which suggests that the estimates of the mean weighted effect sizes were not likely to be biased. One distribution of effect sizes that does appear asymmetrical, however, is that of the relationship between the FWHR and threat, with many smaller samples producing larger positive effect sizes. Although the fail-safe *n*s associated with these analyses indicate that the relationship was robust, the skew in the effect sizes suggests that the estimate for the mean weighted effect size for the FWHR-threat relationship may have been biased by the results of these smaller studies.

## Discussion

Our meta-analyses addressed many outstanding discrepancies in the literature on the FWHR, and confirm its relationship with threat and dominant behaviour (a robust, albeit small, effect size) and with observers’ judgements of these traits (robust, and larger effect sizes). Studies of the FWHR were propelled by Weston and colleagues’ [25] report that this metric was sexually dimorphic (men > women), independent of body size, and emerged at puberty coincident with the rise in androgens. Despite several failures to replicate the sex difference [23,24], our meta-analysis revealed a small but significant sex difference in the FWHR, with men’s FWHRs slightly larger than women’s. Further, the meta-analysis indicated a positive association between judgements of masculinity and the FWHR in men. Although the independence of the FWHR to allometric scaling has not been tested further, studies have investigated the relationship between body mass index (BMI) and the FWHR (e.g., [60,61]), which our meta-analysis indicated was moderately associated with the FWHR in both sexes. Although this relationship with BMI may explain some of the association between the FWHR and behaviour [60], in several studies the relationships were similar when controlling for BMI and when BMI was not controlled (e.g., [17,27,56,62]).

Weston and colleagues [25] speculated that sexual dimorphism in the FWHR evolved via female choice as an attractive trait. Although studies have reported positive associations between the FWHR and male reproductive success (e.g., [21,54]), the meta-analysis found that wider-faced men are judged as less attractive, especially by women, than are narrow-faced men. However, body size and androgen-dependent traits also function in intra-sexual competition [63]. Indeed, our meta-analyses found that men with relatively larger FWHRs behaved in more threatening ways and described themselves as more aggressive, uncooperative, and prejudiced than did men with smaller FWHRs. Further, the FWHR strongly cued judgements of threat, and particularly judgements of aggressiveness as opposed to other indices of threat (e.g., untrustworthiness, prejudice), especially in younger faces. Sensitivity to the FWHR may be enhanced in younger male faces because young men have higher rates of violence and aggression than do the other demographic groups [64,65]. Likewise, the small but significant relationship between the larger FWHRs and dominance mirrored those for threat behaviour and were largely driven by men. The FWHR also cued dominance, but only for judgements of male faces. Although samples are predominantly restricted to studies among men, the small positive correlations between the FWHR and measures of athletic performance and success in business we found suggest a role of intra-sexual competition in shaping sex differences in the FWHR. In the dominance literature, several researchers have distinguished between social and physical forms of dominance (e.g., [66,67]): individuals high in social dominance tend to be influential, respected, and a leader, whereas individuals high in physical dominance tend to be more capable of winning physical fights or contests against other members of the same sex. Most measures used in our meta-analysis of dominance involved questionnaires that captured better the construct of social rather than physical dominance, precluding our ability to formally test whether the type of dominance moderated the relationship. Similarly, in our analysis on the relationship between the FWHR and perceptions of dominance, only two of the included studies explicitly tapped into social dominance (the studies involved judgements of leadership and of social power, see S2 File), whereas other studies did not specify the type of dominance judgements they obtained. Further, one of the two studies produced an outlying effect size that was removed from the final analysis, again precluding our ability to test the type of dominance as a moderator. Future studies investigating the link between FWHR and perceived or actual dominance may benefit from including measures of both social and physical forms or judgements of dominance (e.g., see [57,68]).

The FWHR is not the sole facial metric associated with masculine dominance and aggressiveness; studies have implicated jaw width [69], brow height, eye length, and mouth width [70]. However, the FWHR is well-situated in the upper face, where humans preferentially extract information about threat [71]. Further, whereas dominance and aggressiveness ratings of features such as jawlines may become enhanced by facial hair [72], the association between ratings of aggressiveness and the FWHR is independent of beardedness [13]. Perception of the FWHR involves low spatial frequency processing [12], as do social judgements [73], and thus the FWHR is perceived over longer distances than are specific facial features that rely on high spatial frequency processing. Low spatial frequency processing is rapid, as is assessment of the FWHR [12]. Whether the FHWR cues judgements of threat and dominance because it subtly resembles angry expressions (e.g., the overgeneralization of emotional expression hypothesis [74]) or because the emotional expression of anger simulates social cues of dominance and threat [75] that become amplified by the FWHR remains to be determined. Nevertheless, our meta-analyses provide a starting point for addressing the hypothesis that the FWHR is part of an evolved cueing system of intra-sexual threat, dominance, and aggressiveness in men.

## Supporting Information

S1 File Supplementary Materials and methods. (DOCX)

S2 File Supplementary Tables A through K showing the effect sizes included in each analysis. (DOCX)

S3 File PRISMA Checklist. (DOC)

S1 Fig Funnel plots. (TIF)
