# Supplementary material for: Evidence from Meta-Analyses of the Facial Width-to-Height Ratio as an Evolved Cue of Threat
Source: PLoS One. 2015 Jul 16;10(7):e0132726. doi: 10.1371/journal.pone.0132726 (PMC4504483; doi:10.1371/journal.pone.0132726)
Supplement: S1 File — (DOCX) [file pone.0132726.s001.docx]

**Supplementary Materials and methods**

**Additional notes regarding data extraction and effect size calculations**

If more than one effect size was presented in a paper (e.g., one analysis included a covariate and another analysis did not), we used the effect size from the analysis with the fewest covariates (unless otherwise stated), thus providing the most unbiased estimate of the bivariate association between the FWHR and the variable of interest. If there were many tests of the same hypothesis using a variety of metrics that were conceptually related (explicit prejudice, implicit prejudice, motivation to respond without prejudice, [1]; self-reported dominance, self-reported prestige, Study 3 of [2]), we averaged the effect sizes from the study (if the *n*s used for the effects differed, we used a weighted mean). When two analyses of the same data were conducted within a data set (a correlation versus a median split and t-test), we used results from the analysis that utilized more of the data (e.g., correlations utilize more of the data array than do t-tests on a variable that has been split into low and high groups based on the median score). In studies investigating the relationship between the FWHR and social judgements, we used the effect sizes derived from analyses at the level of the stimuli rather than at the level of the individual. Specifically, some studies examine the association between the mean ratings of faces (averaged across multiple observers) and the FWHRs of the faces. Other studies examine the association between each individual observers’ rating and the face ratio. If both levels of analysis were provided in a manuscript, we used the effect size from the analysis at the level of stimuli, not at the level of the observers. We also averaged the effect sizes when two highly correlated social judgements were collected from the same faces (e.g., trustworthiness and aggression, toughness and aggression).

If a given descriptive statistic (e.g., age) was reported for the sexes combined, we assumed the values to be equal in both sexes. If descriptive statistics were provided separately for men and women and we combined the sexes in an analysis, we calculated the weighted mean. If the number of men and women included in an analysis was not reported, we assumed the sexes to be equally represented (and rounded up for non-discrete numbers). When age was not reported for an effect in a college/university sample, we assumed an age of 20. If the mean age was not reported but an age range was reported, we used the midpoint of the range as the mean age of the sample. If the authors used a university/college sample, nationality was inferred based on the location of the university/college. For studies that examined sex differences in the FWHR and reported results using a variety of facial measures, we preferentially used measures obtained from 2D photos for consistency with other studies. Studies providing only 3D measures and no 2D measures (e.g., anthropometric, 3D scans, dry skulls, [3–5]) were included.

To investigate the link between FWHR and social judgements, some studies have used a continua of faces and a correlational design (examined associations between FWHRs of a variety of faces and the social judgements made for each of the faces), whereas others have compared the judgements of two or more versions of a face, with one of the versions morphed to have a larger FWHR and the other morphed to have a smaller FWHR. Results from these two types of studies were analyzed separately because of the different calculations used to determine the confidence intervals for each type of study; whereas the confidence intervals of studies with a correlational design depended on the number of faces used as stimuli, the confidence intervals of studies with an experimental design depended on the number of observers.

**References**

1. Hehman E, Leitner JB, Deegan, MP, Gaertner SL. Facial structure is indicative of explicit support for prejudicial beliefs. Psychol Sci. 2013; 24: 289–296. doi:10.1177/0956797612451467

2. Mileva VR, Cowan ML, Cobey K D, Knowles KK, Little AC. In the face of dominance: Self-perceived and other-perceived dominance are positively associated with facial-width-to-height ratio in men. Pers Individ Diff. 2010; 69: 115–118. doi:10.1016/j.paid.2014.05.019

3. Weston EM, Friday AE, Liò P. Biometric evidence that sexual selection has shaped the hominin face. PLOS ONE. 2007; 2:e710. doi:10.1371/journal.pone.0000710

4. Gómez-Valdés J. et al. Lack of support for the association between facial shape and aggression: a reappraisal based on a worldwide population genetics perspective. PLOS ONE. 2013;8: e52317. doi:10.1371/journal.pone.0052317

5. Stirrat M, Stulp G, Pollet TV. Male facial width is associated with death by contact violence: Narrow-faced males are more likely to die from contact violence. Evol Hum Behav. 2012;33: 551–556. doi:10.1016/j.evolhumbehav.2012.02.002
