# Supplementary material for: Evidence from Meta-Analyses of the Facial Width-to-Height Ratio as an Evolved Cue of Threat
Source: PLoS One. 2015 Jul 16;10(7):e0132726. doi: 10.1371/journal.pone.0132726 (PMC4504483; doi:10.1371/journal.pone.0132726)
Supplement: S2 File — (DOCX) [file pone.0132726.s002.docx]

S1 Table. Effects included in the analysis of sex differences in the size of the FWHR.

| Study | Sample Size | | Nationality of Sample | *M*_age_ | Measurement Details | FWHR | | | | Standardized Mean Difference (*d*), adjusted for small sample size bias |
| --- | --- | --- | --- | --- | --- | --- | --- | --- | --- | --- |
|  | Men | Women |  |  |  | Men | | Women | |  |
|  |  |  |  |  |  | *M* | *SD* | *M* | *SD* |  |
| Carré & McCormick, [1], Study 1 | 37 | 51 | USA | 18.98 | 2D Photo | 1.860 | 0.130 | 1.800 | 0.100 | **0.496** |
| Carré, Murphy, & Hariri, [2] | 27 | 36 | USA | 19.39 | 2D Photo | 1.810 | 0.120 | 1.750 | 0.120 | 0.494 |
| Geniole, Keyes, Carré, & McCormick, [3] | 146 | 76 | North America | 20.28 | 2D Photo | 1.790 | 0.160 | 1.800 | 0.120 | -0.068 |
| Goetz, Shattuck, Miller, Campbell, Lozoya, Weisfeld, & Carré, [4], Study 1 | 106 | 113 | USA | 21.75 | 2D photo | 1.718 | 0.122 | 1.703 | 0.148 | 0.110 |
| Gómez-Valdés et al., [5], 2D database | 302 | 278 |  |  | 2D Cranial Landmark Coordinates |  |  |  |  | 0.145 |
| Gómez-Valdés et al., [5], 3D database | 401 | 381 |  |  | 3D Cranial Landmark Coordinates |  |  |  |  | 0.003 |
| Gómez-Valdés et al., [5], Hallstat database | 179 | 117 | Austria |  | 3D Cranial Landmark Coordinates |  |  |  |  | **0.362** |
| Gómez-Valdés et al., [5], HOWELLS database | 2256 | 1156 | Int |  | Cranial Measures |  |  |  |  | 0.061 |
| Gómez-Valdés et al., [5], Patagonia database | 111 | 149 | Argentina |  | Cranial Measures |  |  |  |  | 0.043 |
| Gómez-Valdés et al., [5], Pucciarelli database | 297 | 143 |  |  | Cranial Measures |  |  |  |  | 0.191 |
| Haselhuhn & Wong, [6], Study 1 | 51 | 45 | USA | 28 | 2D Photo | 1.780 | 0.120 | 1.740 | 0.120 | 0.330 |
| Haselhuhn & Wong, [6], Study 2 | 50 | 53 | USA | 22 | 2D Photo | 1.790 | 0.150 | 1.730 | 0.210 | 0.325 |
| Hehman, Leitner, & Gaertner, [7], Study 2 | 10 | 10 | USA | 20 | 2D photo | 1.610 | 0.070 | 1.520 | 0.080 | **1.140** |
| Huh, [8] | 64 | 45 | Republic of Korea | 21.73 | 2D Photo | 1.660 | 0.170 | 1.380 | 0.300 | **1.197** |
| Huh, Yi, & Zhu, [9] | 50 | 47 | Republic of Korea | 31.79 | 2D Photo | 1.780 | 0.130 | 1.780 | 0.110 | 0.000 |
| Denson, unpublished | 42 | 84 | Australia |  | 2D Photo | 1.861 | 0.1220 | 1.800 | 0.119 | **0.505** |
| Kramer, Jones, & Ward, [10], Study 1 | 138 | 227 | German | 24 | 2D Photo | 1.850 | 0.110 | 1.870 | 0.110 | -0.182 |
| Kramer, Jones, & Ward, [10], Study 2 | 66 | 89 | UK | 24 | 2D Photo | 2.010 | 0.160 | 2.030 | 0.140 | 0.133 |
| Kramer, Jones, & Ward, [10], Study 3 | 75 | 105 | UK | 23.5 | 2D Photo | 2.070 | 0.160 | 2.070 | 0.150 | 0.000 |
| Lefevre, Etchells, Howell, Clark, & Penton-Voak, [11] | 54 | 49 | UK | 21.59 | 2D Photo | 2.080 | 0.170 | 2.010 | 0.130 | **0.457** |
| Lefevre, Lewis, Bates, Dzhelyova, Coetzee, Deary, & Perrett, [12], Sample 1 | 46 | 99 | UK | 20.22 | 2D Photo | 2.120 | 0.182 | 2.170 | 0.157 | -0.301 |
| Lefevre, Lewis, Bates, Dzhelyova, Coetzee, Deary, & Perrett, [12], Sample 2 | 137 | 169 | UK | 83 | 2D Photo | 2.060 | 0.170 | 2.090 | 0.164 | -0.180 |
| Lefevre, Lewis, Bates, Dzhelyova, Coetzee, Deary, & Perrett, [12], Sample 3 | 124 | 131 | UK | 20.34 | 3D Photo | 1.840 | 0.127 | 1.880 | 0.114 | **-0.331** |
| Lefevre, Lewis, Bates, Dzhelyova, Coetzee, Deary, & Perrett, [12], Sample 4 | 108 | 110 | South Africa | 20.22 | 2D Photo | 2.200 | 0.237 | 2.240 | 0.204 | -0.180 |
| Mileva, Cowan, Cobey, Knowles, & Little, [13], Study 1 | 50 | 50 | UK | 20.6 | 2D Photo | 1.960 | 0.141^a^ | 1.960 | 0.141^a^ | 0.000 |
| Mileva, Cowan, Cobey, Knowles, & Little, [13], Study 2 | 31 | 29 | UK | 21.9 | 2D Photo | 1.980 | 0.111^a^ | 1.960 | 0.162^a^ | 0.143 |
| Mileva, Cowan, Cobey, Knowles, & Little, [13], Study 3 | 21 | 29 | UK | 20.5 | 2D Photo | 1.960 | 0.138^a^ | 1.950 | 0.162^a^ | 0.065 |
| Özener, 2012, Study 1 | 230 | 240 | Turkey | 20.8 | 2D Photo | 1.890 | 0.120 | 1.910 | 0.110 | -0.174 |
| Sanchez-Pages, Rodriguez-Ruiz, & Turiegano, [14] | 147 | 74 | Spain and UK | 20.34 | 2D Photo | 2.090 | 0.158^a^ | 2.047 | 0.129^a^ | **0.288** |
| Skorska, Geniole, Vrysen, McCormick, & Bogaert, [15] | 204 | 186 | Canada | 24.37 | 2D Photo | 1.951 | 0.155 | 1.955 | 0.138 | -0.027 |
| Stirrat, Stulp, Pollet, [16] | 523 | 339 | USA | 36.76 | Skull Measures | 1.824 | 0.110 | 1.821 | 0.110 | 0.027 |
| Weston, Friday, & Liò, [17], Supporting Table S2^b^ | 30 | 30 | South Africa | 23.69 | Coordinates from Dried Skulls | 1.920 | 0.100 | 1.840 | 0.100 | **0.841** |

Effect sizes in bold were reported as significant in the corresponding manuscripts. USA = United States of America. UK = United Kingdom. Int = International sample.

^a^These values were calculated by multiplying the SEM by the square root of n.

^b^Additional data from other age groups were plotted as medians and quartiles, thus means and standard deviations were not extractable. Nevertheless, the sexual dimorphism in the FWHR is thought to emerge after puberty.

S2 Table. Effects included in the analysis of the relationship between the FWHR and perceptions of masculinity, restricted to studies using a correlational design and/or a continuum of faces with un-manipulated FWHRs.

| Study | Observers | | | |  | Stimuli | | | | | Effect size (*r*)^a^ |
| --- | --- | --- | --- | --- | --- | --- | --- | --- | --- | --- | --- |
|  | Men | Women | Nationality | *M*_age_ |  | Men | Women | Nationality | *M*_age_ | Display Details |  |
| Boshyan et al., [18] Study 2 | 8 | 8 | USA | 75.6 |  | 24 |  | USA | 19.08 | 3000 ms, B/W | **.410** |
| Boshyan, et al., [18] Study 2 | 8 | 8 | USA | 18.8 |  | 24 |  | USA | 19.08 | 3000 ms, B/W | .300 |
| Carré, McCormick, & Mondloch [19], Study 1 | 15 | 16 | Canada | 19.94 |  | 24 |  | USA | 19.08 | 2000 ms, B/W | **.430** |
| Geniole & McCormick, [20], Study 1 |  | 29 | Canada | 19.41 |  | 25 |  | Canada | 19.52 | 1000 ms, B/W | **.420** |
| Geniole & McCormick, [20], Study 1 |  | 29 | Canada | 19.41 |  | 54 |  | Canada | 20.32 | 1000 ms, Facegen, B/W | **.500** |
| Geniole & McCormick, [20], Study 2 |  | 26 | Canada | 20.69 |  | 54 |  | Canada | 20.32 | 1000 ms, Facegen, B/W | **.280** |
| Geniole & McCormick, [21] | 22 | 34 | Canada | 19.89 |  | 25 |  |  |  | B/W, non-bearded faces^b^ | **.550** |
| Geniole, Keyes, Mondloch, Carré, & McCormick, [22], Study 1 | 10 | 10 | Canada | 20.63 |  | 24 |  | USA | 19.08 | 2000 ms, B/W | **.460** |
| Geniole, Keyes, Mondloch, Carré, & McCormick, [22], Study 1 | 10 | 10 | Canada | 20.63 |  |  | 31 | USA | 18.87 | 2000 ms, B/W | -.050 |
| Geniole, Keyes, Mondloch, Carré, & McCormick, [22], Study 2 | 10 | 10 | Canada | 22.3 |  |  | 31 | USA | 18.87 | 2000 ms, B/W | .040 |
| Geniole, Keyes, Mondloch, Carré, & McCormick, [22], Study 3 | 20 | 20 | Canada | 19.95 |  | 24 |  | USA | 19.08 | 2000 ms, B/W | .360 |
| Sanchez-Pages, Rodriguez-Ruiz, & Turiegano, [14] | 36 |  |  | 31.17 |  | 147 |  | UK, Spain | 20.34 |  | .081 |

Effects were from studies examining judgements of masculinity or of femininity (which we reversed). Effect sizes in bold were reported as significant in the corresponding manuscripts. USA = United States of America. UK = United Kingdom. Int = International sample.

^a^positive values indicate that individuals with larger FWHRs are judged as more masculine (or less feminine) than individuals with smaller FWHRs.

^b^the authors also examined the relationship between the FWHR and judgements of masculinity in bearded versions of the same male faces (*r* = .07, *p* = .71). We chose to only include effects from the non-bearded versions of the faces given most perceptions studies involving the FWHR have used non-bearded faces as stimuli. We note that after averaging the effect sizes, however, the mean weighted effect size is still significant (*k* = 12, $\bar{r}$ = .28, *95% CI* = .16 to .39, *p* < .0001; *Q*_11_ = 16.81, *p* = .11).

S3 Table. Effects included in the analysis on the relationship between the FWHR and threat behaviour.

| Study | Sample Size | | Nationality of Sample | *M*_age_ | Measure | Type of Measure | Type of Threat | Effect Size (*r*)^a^ |
| --- | --- | --- | --- | --- | --- | --- | --- | --- |
|  | Men | Women |  |  |  |  |  |  |
| Carré & McCormick, [1], Study 1 | 37 |  | USA | 18.98 | Point Subtraction Aggression Paradigm [23] | Behaviour | Aggression | **.380** |
| Carré & McCormick, [1], Study 1 |  | 51 | USA | 18.98 | Point Subtraction Aggression Paradigm [23] | Behaviour | Aggression | -.045^b^ |
| Carré & McCormick, [1], Study 2 | 21 |  | Canada | 20 | Penalty minutes per game in varsity hockey players | Behaviour | Aggression | **.540** |
| Carré & McCormick, [1], Study 3 | 112 |  | Int |  | Penalty minutes per game in NHL players during 2007-2008 season | Behaviour | Aggression | **.300** |
| Carré, Murphy, & Hariri, [2] | 27 |  | USA | 19.39 | Physical Aggression Subscale of the Buss-Perry Aggression Questionnaire [24] | Self-report | Aggression | -.040^b^ |
| Carré, Murphy, & Hariri, [2] |  | 36 | USA | 19.39 | Physical Aggression Subscale of the Buss-Perry Aggression Questionnaire [24] | Self-report | Aggression | .014^b^ |
| Deaner, Goetz, Shattuck, & Schnotala, [25] | 520 |  | Int |  | Career penalty minutes per game of NHL players on 2011-2012 rosters | Behaviour | Aggression | .084^c^ |
| Efferson & Vogt, [26] | 41 |  | Germany | 20 | Tendency to exploit another player’s trust | Behaviour | Selfish and Pejorative | .369 |
| Geniole, Keyes, Carré, & McCormick, [3] | 146 |  | North America | 20.28 | Average of tendency to cheat (***r* = .241**) and extent of cheating (***r* = .230**) in a lottery for a cash prize | Behaviour | Selfish and Pejorative | .240^ƚ^ |
| Geniole, Keyes, Carré, & McCormick, [3] |  | 76 | North America | 20.28 | Average of tendency to cheat (*r* = -.042) and extent of cheating (*r* = .147) in a lottery for a cash prize | Behaviour | Selfish and Pejorative | .053^ƚ^ |
| Goetz, Shattuck, Miller, Campbell, Lozoya, Weisfeld, & Carré, [4], Study 1 | 108 |  | USA | 21.75 | Point Subtraction Aggression Paradigm [23] | Behaviour | Aggression | **.187** |
| Goetz, Shattuck, Miller, Campbell, Lozoya, Weisfeld, & Carré, [4], Study 1 |  | 113 | USA | 21.75 | Point Subtraction Aggression Paradigm [23] | Behaviour | Aggression | -.008^b^ |
| Goetz, Shattuck, Miller, Campbell, Lozoya, Weisfeld, & Carré, [4], Study 2 | 868 |  | Int |  | Penalty minutes per game in NHL players during 2010-2011 | Behaviour | Aggression | **.080** |
| Gómez-Valdés et al., [5] | 163 |  | Mexico |  | Difference in the size of the FWHR between the general population and weighted mean of criminal groups^d^ | Behaviour | Selfish and Pejorative | -.341^e^ |
| Haselhuhn & Wong, [6], Study 1 | 51 |  | USA | 28 | Explicit deception during negotiation in the Bullard House negotiation exercise [27] | Behaviour | Selfish and Pejorative | **.290** |
| Haselhuhn & Wong, [6], Study 1 |  | 45 | USA | 28 | Explicit deception during negotiation in the Bullard House negotiation exercise [27] | Behaviour | Selfish and Pejorative | -.166 |
| Haselhuhn & Wong, [6], Study 2 | 50 |  | USA | 22 | Inferred cheating in a lottery | Behaviour | Selfish and Pejorative | **.360** |
| Haselhuhn & Wong, [6], Study 2 |  | 53 | USA | 22 | Inferred cheating in a lottery | Behaviour | Selfish and Pejorative | -.010 |
| Haselhuhn, Wong, & Ormiston, [28], Study 1 | 131 |  | UK | ~26^f^ | Tendency for a pro-self rather than a prosocial resource allocation strategy | Behaviour | Selfish and Pejorative | **.180** |
| Haselhuhn, Wong, Ormiston, Inesi, & Galinsky, [29], Study 2 | 30 same-sex dyads |  | USA | 20 | Self-reported cooperativeness (reverse coded) in negotiation | Self-report | Selfish and Pejorative | **.410** |
| Hehman, Leitner, Deegan, & Gaertner, [30], Study 1 | 66^g^ |  | USA | 20 | Average of the relationships between the FWHR and explicit prejudice, controlling for implicit prejudice **(***n* = 70, ***sr* = .211)**, implicit prejudice controlling for explicit prejudice (*n* = 70, *pr* = -.038), and motivation to respond without prejudice **(reversed;** *n* = 57, ***r =* .295)** | Self-report^h^ | Selfish and Pejorative | .147^ƚ^ |
| Jia et al., [31] | 720 |  | Int |  | CEOs risk of financial misreporting (see F-Risk values in Table 3, panel B of Jia et al., [31]) | Behaviour | Selfish and Pejorative | **.045** |
| Lefevre, Etchells, Howell, Clark, & Penton-Voak, [11] | 54 |  | UK | 21.59 | Buss-Perry Aggression Questionnaire [24] | Self-report | Aggression | **.270** |
| Lefevre, Etchells, Howell, Clark, & Penton-Voak, [11] |  | 49 | UK | 21.59 | Buss-Perry Aggression Questionnaire [24] | Self-report | Aggression | .170 |
| Özener, [32], Study 2 | 108 |  | Turkey | 20.05 | Aggression Questionnaire (34 items; Buss & Warren, [33]) | Self-report | Aggression | -.001 |
| Özener, [32], Study 2 |  | 104 | Turkey | 20.74 | Aggression Questionnaire (34 items; Buss & Warren, [33]) | Self-report | Aggression | .051 |
| Stirrat & Perrett, [34] | 36 |  | UK | 21.6 | Tendency to exploit the trust of another person for financial gain | Behaviour | Selfish and Pejorative | **.400** |
| Stirrat & Perrett, [34] |  | 107 | UK | 21.6 | Tendency to exploit the trust of another person for financial gain | Behaviour | Selfish and Pejorative | .160 |
| Stirrat & Perrett, [35] | 17 |  | UK | 20.6 | Uncooperative behaviour in a public goods game | Behaviour | Selfish and Pejorative | **.500** |
| Třebický, Fialová, Kleisner, Roberts, Little, & Havlíček, [36] | 146 |  | Int | 29.77 | UFC Performance: proportion of wins to fights | Behaviour | Aggression | **.114** |
| Welker, Goetz, Galicia, Liphardt, & Carré, [37] | 910 |  | Int |  | Total fouls committed (excluding offsides) by football players in the 2010 World Cup | Behaviour | Aggression | .057 |
| Zilioli, Sell, Stirrat, Jagore, Vikckerman, & Watson, [38], Study 1 | 241 |  | Int |  | UFC performance: Average of FWHR correlations with total fights **(*r* = .163**), number of wins **(*r* =** **.203**), and win percentage controlling for number of fights (***r* =** **.139**)^i^ | Behaviour | Aggression | .168^ƚ^ |

Effect sizes in bold were reported as significant in the corresponding manuscripts.

^ƚ^Effect size represents an average for which we did not determine statistical significance. USA = United States of America. Int = International. UK = United Kingdom. NHL = National Hockey League. ^a^positive correlations indicate that individuals with larger FWHRs had greater values on the DV of interest (i.e., more threat behaviour) than those with smaller FWHRs.

^b^data obtained from one of the current manuscript’s authors.

^c^Although the authors also provide penalty minutes related to fighting, we chose to use overall penalty minutes for consistency with other studies [1,4] and because it may more broadly represent the construct of aggression than penalty minutes specific to fighting. Also, the authors focused on penalty minutes to test their main hypothesis.

^d^General population (*n* = 56; mean = 1.908, *SD* = 0.125) and weighted mean and pooled *SD* of criminal groups (homicide: *n* = 58, mean = 1.838, *SD* = 0.118; robbery: *n* = 42, mean = 1.809, *SD* = 0.114; other minor fault: *n* = 7, mean = 1.765, *SD* = 0.089; total *n* of criminal groups = 107, weighted mean of criminal groups = 1.822 pooled *SD* of criminal groups = 0.115). Positive correlation value indicates a larger FWHR among criminals than the general population.

^e^effect size calculated after estimating means and standard deviations from Figure 2b in Gómez-Valdés et al. [5].

^f^Authors did not collect information about age but drafted from a pool of students that had a mean age of 26.

^g^This n was calculated by averaging the number of participants included in each of three analyses performed by the authors (average *n* = 66).

^h^Although this measure involved an Implicit Associations Test as one of the measures of prejudice, the other two measures were self-report and thus we classified this effect as self-report rather than behavioural.

^i^we use an effect size involving a covariate here because the correlation between the FWHR and win percentage (without controlling for number of fights) was driven by fighters with relatively few fights [38].

S4 Table. Effects included in the analysis on the relationship between the FWHR and dominance.

| Study | Sample Size | | | Nationality of Sample | *M*_age_ | Measure | Effect Size (*r*)^a^ |
| --- | --- | --- | --- | --- | --- | --- | --- |
|  | Men | Women | |  |  |  |  |
| Carré & McCormick, [1] | 37 | |  | USA | 18.98 | Trait dominance (10 item International Personality Item Pool scale, IPIP, [39]) | -.063 |
| Carré & McCormick, [1] |  | | 51 | USA | 18.98 | Trait dominance (10 item IPIP, [39]) | .026 |
| Geniole, Keyes, Carré, & McCormick, [3] | 146 | |  | North America | 20.28 | Fearless Dominance measured by the Psychopathic Personality Inventory-Revised [40] | .151^b^ |
| Geniole, Keyes, Carré, & McCormick, [3] |  | | 76 | North America | 20.28 | Fearless Dominance measured by the Psychopathic Personality Inventory-Revised [40] | .075^b^ |
| Haselhuhn & Wong, [6], Study 2 | 50 | |  | USA | 22 | Psychological sense of power [41] | **.310** |
| Haselhuhn & Wong, [6], Study 2 |  | | 53 | USA | 22 | Psychological sense of power [41] | .170 |
| Haselhuhn, Wong, Ormiston, Inesi, & Galinsky, [29], Study 2 | 30 same-sex dyads (60 men) | |  | USA | 20 | Self-reported competitiveness in negotiation (How competitive do you intend to be in the upcoming negotiation, 1=not at all competitive, 7 = extremely competitive) | −.050 |
| Lefevre, Etchells, Howell, Clark, & Penton-Voak, [11] | 54 | |  | UK | 21.59 | Dominance (11 item IPIP, [39]) | .**290** |
| Lefevre, Etchells, Howell, Clark, & Penton-Voak, [11] |  | | 49 | UK | 21.59 | Dominance (11 item IPIP, [39]) | .120 |
| Lefevre, Lewis, Perrett, & Penke, [42], Sample 1, supplementary data | 185 | |  | Germany | 33.61 | Competitiveness: average of the FWHRs correlations with competitiveness (*r* = .080) and with tendency to experience others as same-sex rivals (*r* = .011) | .046^ƚ^ |
| Lewis, Lefevre, & Bates, [43] | 29^c^ | |  | USA |  | President’s dominance as an average of the FWHRs correlations with achievement drive (*n* =28, ***r* = .580**), forcefulness (*n* = 29, *r* = .130), and inflexibility (*n* = 29, *r* = .170) | .290^ƚ, c^ |
| Loehr & O’Hara, [44] | 795 | |  | Finland |  | Military rank at start of war (enlisted, junior officer, senior officer) | **-.152**^d^ |
| Mileva, Cowan, Cobey, Knowles, & Little, [13], Study 2 | 31 | |  | UK | 21.9 | Trait dominance (IPIP; [45]) 11 items plus an additional question: “I get my own way” | **.450** |
| Mileva, Cowan, Cobey, Knowles, & Little, [13], Study 2 |  | | 29 | UK | 21.9 | Trait dominance (IPIP; [45]) 11 items plus an additional question: “I get my own way” | .160 |
| Mileva, Cowan, Cobey, Knowles, & Little, [13], Study 3 | 21 | |  | UK | 20.5 | Self-reported dominance [average of dominance (***r* =** **.510**) and prestige (*r* = -.020)] (Prestige-Dominance Questionnaire, [46]) | .245^ƚ^ |
| Mileva, Cowan, Cobey, Knowles, & Little, [13], Study 3 |  | | 29 | UK | 20.5 | Self-reported dominance [average of dominance (*r* = .030) and prestige (*r* = -.020)] (Prestige-Dominance Questionnaire, [46]) | .005^ƚ^ |
| Valentine, Li, Penke, & Perrett, [47] | 78 | |  | Germany | 26.5 | Assured-dominant and unassured-submissive (reverse-scored) (German Revised Interpersonal Adjective Subcales, [48]) controlling for adiposity | -.050 |

Effect sizes in bold were reported as significant in the corresponding manuscript. When analyses were conducted at the level of a dyad rather than at the level of the individual, we counted each dyad as one participant. USA = United States of America. UK = United Kingdom. IPIP = International Personality Item Pool.

^ƚ^Effect size represents an average for which we did not determine statistical significance.

^a^positive values indicate that individuals with larger FWHRs were more dominant on the corresponding measure than were individuals with smaller FWHRs.

^b^these bivariate effects were not reported in the paper but are provided here by authors.

^c^These values reflect the average n and the effect size weighted by the number of participants.

^d^we accessed the online database to obtain this bivariate correlation.

S5 Table. Effects included in the analysis of the relationship between the FWHR and success in business-related outcomes.

| Study | Sample Size | Nationality of Sample | *M*_age_ | Measure | Effect Size (*r*)^a^ |
| --- | --- | --- | --- | --- | --- |
|  | Men |  |  |  |  |
| Alrajih & Ward, [49] | 186 | UK | 52.5 | CEO (*n* = 93) vs controls matched on ethnicity and (approximate) age (*n* = 93) | **.481** |
| Yang, Chao, Fabiansson, & Denson, unpublished manuscript | 86 groups (244 individuals) | China | 20 | Value claimed in negotiation | .060 |
| Haselhuhn, Wong, Ormiston, Inesi, & Galinsky, [29], Study 1 | 23 same-sex dyads (46 men) | USA | 20 | Seller’s negotiation performance (sale price) in 23 same sex dyads (*n* = 46) | **.430**^b^ |
| Haselhuhn, Wong, Ormiston, Inesi, & Galinsky, [29], Study 2 | 30 same-sex dyads (60 men) | USA | 20 | Negotiation for a signing bonus | **.420**^c^ |
| Haselhuhn, Wong, Ormiston, Inesi, & Galinsky, [29], Study 3 | 34 same- sex dyads (70 men)^d^ | USA | 20 | Ability to legitimately (within the rules of the bargaining exercise) come to an agreement between buyer (who can’t afford price) and seller (who can’t make price any lower). | **-.355**^e^ |
| Wong, Ormiston, & Haselhuhn, [50] | 55 | USA | 55.7 | CEO’s return on assets for corresponding firm (average of 2003 and 2004) | .230 |

Effect sizes in bold were reported as significant in the corresponding manuscripts. When analyses were conducted at the level of a dyad rather than at the level of the individual, we counted each dyad as one participant. USA = United States of America. UK = United Kingdom. This analysis does not include effects from Jia et al. [31] given difficulty and inconsistency among the coders in determining which effects should be included in the analysis and difficulty in determining the construct each measure represented. A study by Mayew, Parsons, and Venkatachalam [51] was not included because the authors did not provide numerical values. ^a^Positive numbers indicate that individuals with larger FWHRs perform better than individuals with smaller FWHRs. ^b^This effect reflects the total FWHR size (seller FWHR/ buyer FWHR + seller FWHR). ^c^(candidate FWHR / candidate + recruiter FWHR) higher number indicates candidate has higher FWHR than recruiter. ^d^two men were assigned to play the same role (both played as one member of one of the dyadic interactions. ^e^This effect size was obtained by converting the χ^2^ value to an *r* given that other conversion techniques returned unrealistically high *r* values (*r*s > .90).

S6 Table. Effects included in the analysis of the relationship between the FWHR and sports performance.

| Study | Sample Size | Nationality of Sample | *M*_age_ | Measure | Effect Size (*r*)^a^ |
| --- | --- | --- | --- | --- | --- |
|  | Men |  |  |  |  |
| Třebický, Fialová, Kleisner, Roberts, Little, & Havlíček, [36] | 146 | Int | 29.77 | UFC Performance: proportion of wins to fights | **.114** |
| Tsujimura & Banissy, [52], Study 1 | 104 | Japan | 28.91 | Baseball batting performance in Japanese Central League Pennant baseball (2011). Average of FWHR correlations with batting average (*r* = .171), number of home runs (***r* = .250**), slugging percentage (***r* =** **.206**), hits (*r* = .112), runs-batted-in (*r* = .176), and on base percentage (*r* = .137). | **.**175^ƚ^ |
| Welker, Goetz, Galicia, Liphardt, & Carré, [37], defenders, midfielders, forwards | 910 | Int |  | Average of correlation between FWHR and total goals (*r* = .026) and of FWHR and total assists (*r* = .002) controlling for total games played, height, weight, fouls committed against the player, and player position (defender, midfielder, forward) | .014^ƚ^ |
| Welker, Goetz, Galicia, Liphardt, & Carré, [37], forwards only | 211 | Int |  | Average of correlation between FWHR and total goals (***r* = .152**) and of FWHR and total assists (***r* =** **.136**) among forwards, controlling for total games played, height, weight, fouls committed against the player | .144^ƚ^ |
| Zilioli, Sell, Stirrat, Jagore, Vikckerman, & Watson, [38], Study 1 | 241 | Int |  | UFC fight performance: Average of FWHR correlation with total fights (***r* = .163**), number of wins (***r* = .203**), and win percentage controlling for number of fights (***r* =** **.139**)^b^ | .168^ƚ^ |

Correlations in bold were reported as significant in the corresponding manuscripts.

^ƚ^Effect size represents an average for which we did not determine statistical significance. Int = International sample. UFC = Ultimate Fighting Championships. ^a^positive values indicate that individuals with larger FWHRs have better sports performance than those with lower FWHRs.

^b^we use an effect size involving a covariate here because the correlation between the FWHR and win percentage (without controlling for number of fights) was driven by fighters with relatively few fights.

S7 Table. Effects included in the analysis of the relationship between the FWHR and perceptions of threat, restricted to studies using a correlational design and/or a continuum of faces with un-manipulated FWHRs.

| Study | Observers | | | |  | Stimuli | | | | | Judgement^a^ | Effect Size  (*r*)^b^ |
| --- | --- | --- | --- | --- | --- | --- | --- | --- | --- | --- | --- | --- |
|  | Men | Women | Nation-ality of sample | *M*_age_ |  | Men | Women | Nation-ality of sample | *M*_age_ | Display Details |  |  |
| Alrajih & Ward, [49] | 4^c^ | 6^c^ | UK | 21.5 |  | 153^d^ |  | UK | 52.5 | Colour, 79 CEOs (m actual age = 52.5) and 74 controls (matched ethnicity, age, facial hair, glasses) | A: *r* = .060; T: *r* = .080 | .070^ƚ^ |
| Boshyan, Zebrowitz, Franklin, McCormick, & Carré, [18], Study 2 | 20^e^ | 20^e^ | USA | 18.8^f^ |  | 24 |  | USA | 19.08 | 3000 ms, B/W | A | **.730** |
| Boshyan, Zebrowitz, Franklin, McCormick, & Carré, [18], Study 2 | 18^g^ | 18^g^ | USA | 75.6^f^ |  | 24 |  | USA | 19.08 | 3000 ms, B/W | A | **.470** |
| Carré, McCormick, & Mondloch, [19], Study 1 | 15 | 16 | Canada | 19.94 |  | 24 |  | USA | 19.08 | 2000 ms, B/W | A: ***r* =** **.590**; T: *r* = .450 | .520^ƚ^ |
| Carré, McCormick, & Mondloch, [19], Study 2 and Carré et al., [53], Exp 1B |  | 16 | Canada | 19.38 |  | 24 |  | USA | 19.08 | 39 ms, B/W; 39 ms, B/W, Blurred | A, 39 ms display: ***r* = .700**; A, 39 ms and blurred display: ***r* = .670** | .685^ƚ,h^ |
| Carré, Morrissey, Mondloch, & McCormick, [53], Exp 1A | 8 | 8 | Canada | 23.81 |  | 24 |  | USA | 19.08 | 2000 ms, B/W | A, chin/forehead crop display: ***r* = .790**; A, side crop display: ***r* = .810**; A, Blurred display: ***r* = .670** | .757^ƚ,h^ |
| Carré, Morrissey, Mondloch, & McCormick, [53], Exp 1C | 1 | 9 | Canada | 23.85 |  | 24 |  | USA | 19.08 | 2000 ms, B/W, Blurred | A | **.450** |
| Efferson & Vogt, [26] | 13 | 15 | Ger | 20 |  | 54 |  | Ger |  |  | T | .132 |
| Geniole & McCormick, [20], Study 2^i^ |  | 26 | Canada | 20.69 |  | 54 |  | Canada | 20.32 | 1000 ms, Facegen, B/W | A | **.640** |
| Geniole & McCormick, [21] | 22 | 34 | Canada | 19.89 |  | 25 |  |  |  | B/W, non-bearded faces^j^ | A | **.660** |
| Geniole, Keyes, Mondloch, Carré, & McCormick, [22], Study 1 | 10 | 10 | Canada | 20.63 |  | 24 |  | USA | 19.08 | 2000 ms, B/W | A | **.710** |
| Geniole, Keyes, Mondloch, Carré, & McCormick, [22], Study 1 | 10 | 10 | Canada | 20.63 |  |  | 31 | USA | 18.87 | 2000 ms, B/W | A | **.400** |
| Geniole, Keyes, Mondloch, Carré, & McCormick, [22], Study 2 | 10 | 10 | Canada | 22.3 |  |  | 31 | USA | 18.87 | 2000 ms, B/W | A | **.440** |
| Geniole, Molnar, Carré, & McCormick, [54], footnote #3 | 4 | 36 | Canada | 20.23 |  | 65 |  | North America | 19.30 | 1000 ms display, B/W | A | **.420** |
| Geniole, Molnar, Carré, & McCormick, [54], Study 1 | 5 | 28 | Canada | 20.6 |  | 54 |  | Canada | 20.32 | Facegen, 1000 ms, B/W | A: ***r* = .620**; T: ***r* = .470** | .545^ƚ^ |
| Geniole, Molnar, Carré, & McCormick, [54], Study 1 | 5 | 29 | Canada | 20.6 |  | 25 |  | Canada | 19.52 | 1000 ms, B/W | A: ***r* = .610**; T: ***r* =** **.520** | .565^ƚ^ |
| Geniole, Molnar, Carré, & McCormick, [54], Study 2^k^ | 12 | 12 | Canada | 19.58 |  | 22 |  | Canada | 19.52 | Facial hair, 1000 ms, B/W | A: ***r* = .450**; T: *r* = .280 | .365^ƚ^ |
| Geniole, Molnar, Carré, & McCormick, [54], Study 4 | 8 | 32 | Canada | 19.38 |  | 65 |  | North America | 19.30 | Photos displayed until response was made, B/W | A: ***r* = .480**; T: ***r* = .400** | .440^ƚ^ |
| Hehman, Leitner, & Freeman, [55], Study 3 | 8 | 8 |  |  |  | 60 |  | USA | 45 | 10 white faces from each decade (20s-70s) | F | **.809** |
| Hehman, Leitner, & Gaertner, [7], Study 2 | 51 | 50 | USA | 20 |  | 10 | 10 | USA |  | Head at baseline vs tilted up vs tilted down | A | **.572** |
| Hehman, Leitner, Deegan, & Gaertner, [30], Pilot data, footnote 2 | 25 | 25 | USA | 20 |  | 20^l^ |  | USA^l^ |  |  | P | **.534** |
| Hehman, Leitner, Deegan, & Gaertner, [30], Study 2 | 28^m^ | 74^m^ | USA | 20 |  | 20 |  | USA |  | 5 faces from each quartile of FWHR distribution from Study 1 | P | **.689** |
| Hehman, Leitner, Deegan, & Gaertner, [30], Study 3 | 21^n^ | 26^n^ | USA | 20 |  | 20 |  | USA |  |  | P | **.747** |
| Kleisner, Priplatova, Frost, & Flegr, [56] | 43^o^ | 62^o^ | Czech Republic | 23.1 |  | 40 |  | Czech Republic | 20.8 | Colour | T | **.346** |
| Sanchez-Pages, Rodriguez-Ruiz, & Turiegano, [14] | 11^p^ | 11^p^ |  | 27.48 |  | 147 |  | UK, Spain | 20.34 |  | T | **.339** |
| Short et al., [57] Exp1 | 8 | 8 | Canada | 21.5 |  | 24 |  | USA | 19.08 | 2000 ms, B/W | A | **.640** |
| Short et al., [57] Exp1 | 8 | 8 | Canada | 21.5 |  | 24 |  | China |  | 2000 ms, B/W | A | **.610** |
| Short et al., [57] Exp1 | 8 | 8 | China | 21.5 |  | 24 |  | USA | 19.08 | 2000 ms, B/W | A | **.560** |
| Short et al., [57] Exp1 | 8 | 8 | China | 21.5 |  | 24 |  | China |  | 2000 ms, B/W | A | **.470** |
| Short et al., [57] Exp2 | 8 | 8 | Canada | 8 |  | 24 |  | USA | 19.08 | 2000 ms, B/W | A | **.450** |
| Short et al., [57] Exp2 | 8 | 8 | Canada | 8 |  | 24 |  | China |  | 2000 ms, B/W | A | **.500** |
| Short et al., [57] Exp2 | 8 | 8 | China | 8 |  | 24 |  | USA | 19.08 | 2000 ms, B/W | A | **.460** |
| Short et al., [57] Exp2 | 8 | 8 | China | 8 |  | 24 |  | China |  | 2000 ms, B/W | A | .340 |
| Stirrat & Perrett, [34], Study 2 | 17 | 45 | UK | 20.32 |  | 67 |  | UK | 20.8 |  | T | **.396** |
| Třebický, Fialová, Kleisner, Roberts, Little, & Havlíček, [36] | 216 | 402 | Czech Republic | 26.46 |  | 146 |  | Int | 29.77 |  | A | **.161**^q^ |
| Třebický, Fialová, Kleisner, Roberts, Little, & Havlíček, [36] | 98 | 180 | Czech Republic | 27.53 |  | 146 |  | Int | 29.77 |  | F | **.157**^q^ |
| Valentine, Li, Penke, & Perrett, [47]^r^ | 16 | 15 | Ger | 26.39 |  | 78 |  | Ger | 26.5 |  | A | .150 |
| Zilioli, Sell, Stirrat, Jagore, Vickerman, & Watson, [38], Study 2b | 16 | 16 | Canada | 21.25 |  | 48 |  | Int |  | Rated individuals | F | **.460** |

Effect sizes in bold were reported as significant in the corresponding manuscripts. ^ƚ^Effect size represents an average for which we did not determine statistical significance. We did not include studies if the photos involved faces intentionally posed in non-neutral expressions (e.g., we did not include effects from Marsh and colleagues [58]). USA = United States of America. UK = United Kingdom. Ger = Germany. Int = International sample. A = judgements of aggression. F = judgements of formidability (e.g., How tough does this person look?). T = judgements of trustworthiness, reversed. P = judgements of prejudice. B/W = Black and white. ^a^If multiple effects are provided in a given cell, they were averaged to form a single effect size.

^b^positive values indicate that individuals with larger FWHRs are judged as more threatening than individuals with lower FWHRs. ^c^Sample size of participants based on 8 men, 12 women, who only made three rating types each. Given 6 ratings total, this sample size was estimated to be 4 men and 6 women for each rating.

^d^Although the stimuli was 93 control and 93 CEO faces, thirty-three (14 CEOs) faces were removed because they were recognized by participants.

^e^24 young adults from accuracy condition in Study 1 + 16 new young adults; 8 men, 8 women; sexes assumed to be equally represented.

^f^authors combined multiple samples for this analysis, thus making it difficult to determine the mean age. Here, we report the mean age of the new observers added to the analysis.

^g^35 total (19 old adults from the accuracy condition in Study 1 in addition to 16 new old adults); sexes assumed to be equally represented.

^h^We did not include the correlation obtained from a condition in which the faces were scrambled given this presentation strategy was meant to disrupt the relationship.

^i^Study 1 was not included given it involved a subset of the participants used in Study 1 of Geniole and colleagues [54].

^j^the authors also examined the relationship between the FWHR and judgements of aggression in bearded versions of the same male faces (*r* = .59, *p* = .002). We chose to only include effects from the non-bearded versions of the faces given most perception studies involving the FWHR have used non-bearded faces as stimuli. We note that averaging the effect sizes, however, leads to a similar mean weighted effect size in the final analysis (*k* = 38, $\bar{r}$ = .48, *95% CI* = .41 to .55, *p* < .0001; *Q*_37_ = 124.55, *p* < .0001).

^k^Study 3 of Geniole et al. [54] was a reanalysis of data from Carre et al. [19] and Geniole et al., [22] and was thus not included.

^l^Not reported but assumed the same as Study 2.

^m^one participant of an unspecified sex was removed because he or she recognized one of the stimulus faces. Here we report the *n* before the removal given this ambiguity regarding sex.

^n^two participants of an unspecified sex were removed because they recognized one of the stimulus faces. Here we report the *n* before the removal of the participants given this ambiguity regarding sex.

^o^Number of each sex inferred based on distribution of total sample (142 women, 98 men) from which this subset of 105 was derived. ^p^Although the authors also reported results after splitting the faces into wide and narrow FWHR groups and performing a t-test on the mean differences in judgements between the two groups, we used the effect size based on the correlation between the FWHR and judgements across all faces given this correlation analysis utilizes every data point.

^q^aggressiveness and fighting ability were not averaged in this case because a separate set of raters completed each judgement.

^r^The authors also have the rating “interest in person as a friend”, which we chose not to include here because a lack of interest in friendship does not necessarily imply threat, especially given that the friendship ratings were made in the context of speed dating appraisals.

S8 Table. Effects included in the analysis of the relationship between the FWHR and perceptions of threat, restricted to studies using an experimental design wherein the FWHR was manipulated to appear larger or smaller.

| Study | Observers | | | |  | Stimuli | | | | | Judgement^b^ | St. Mean Dif (*d*), adj^c^ |
| --- | --- | --- | --- | --- | --- | --- | --- | --- | --- | --- | --- | --- |
|  | M | W | Nationality | *M*_age_ |  | M^a^ | W^a^ | Nationality | *M*_age_ | Transformation Details |  |  |
| Bashir & Rule, [59] | 18 | 47 | Canada | 21.34 |  | 1 |  | Canada |  | 1 face, Morphed to have high or low FWHR | T | **-0.566** |
| Hehman, Leitner, & Freeman, [55], Study 1 | 22 | 22 |  |  |  | 30 |  |  | 44 | 30 faces randomly generated and modified in Facegen to look young (~18) middle-aged (~40), and old (~70), each age morphed to a high and low FWHR | A | **3.804** |
| Lefevre & Lewis, [60], Study 1 | 34 | 68 | UK | 25.91 |  | 12 |  | UK |  | 12 composites x 2 prototype sets, transformed 25%, 37.5%, and 50% in shape difference of high and low FWHRs | A | **0.208** |
| Lefevre & Lewis, [60], Study 2 | 68 | 190 | UK | 24.10 |  | 12 |  | UK |  | 12 composites x 1 prototype sets, transformed 37.5% and 50% in shape difference of high and low FWHRs | A, 37.5% transform: **unadjusted *d* = .305**; A, 50% transform: **unadjusted *d* = .468** | 0.386^ƚ^ |
| Lefevre & Lewis, [60], Study 2 | 68 | 190 | UK | 24.1 |  |  | 15 | UK |  | 15 composites x 1 prototype sets, transformed 37.5% and 50% in shape difference of high and low FWHRs | A, 37.5% transform: **unadjusted *d* = 0.524**; A, 50% transform: **unadjusted *d* = 0.643** | 0.582^ƚ^ |
| Stirrat & Perrett, [34], Study 3 | 77 | 208 |  | 23.20 |  | 12 |  | UK | 22.50 | 12 faces x 2 warp directions on FWHR x 3 transformation sets. 3 groups of participants rated each transformation set | T | **0.319** |
| Wang, Geigel, & Herbert, [61] | 37 | 48 |  | 38 |  | 2 |  |  |  | 2 avatars, each warped to have small, medium, and large FWHRs | A | **-0.783** |
| Wang, Geigel, & Herbert, [61] | 37 | 48 |  | 38 |  |  | 2 |  |  | 2 avatars, each warped to have small, medium, and large FWHRs | A | **-0.516** |
| Zilioli, Sell, Stirrat, Jagore, Vickerman, & Watson, [38], Study 2a | 20 | 16 | Canada | 21.74 |  | 15 |  | Int |  | 15 wide, 15 narrow composites | F^d^ | **0.557** |
| Zilioli, Sell, Stirrat, Jagore, Vickerman, & Watson, [38], Study 2a all white faces^e^ | 20 | 20 | Canada | 20.15 |  | 12 |  | Int |  | 12 wide, 12 narrow composites, all white faces | F | **0.794** |
| Zilioli, Sell, Stirrat, Jagore, Vickerman, & Watson, [38], Study 3 | 66 | 58 | Canada | 21.30 |  | 12 |  | Int | 22.50 | Photos from Stirrat & Perrett, 2010 | F | **0.328** |

Effect sizes in bold were reported as significant in the corresponding manuscripts.

St. Mean Dif (*d*), adj = Standardized mean difference, adjusted for small sample size bias.

^ƚ^Effect size represents an average for which we did not determine statistical significance. USA = United States of America. UK = United Kingdom. Int = International sample.

^a^Given ambiguity regarding the exact number of face pairs included in each analysis, especially after we averaged across some effects within a given study, we only report the number of base images or base composites used to create the images.

^b^If multiple effects are provided in a given cell, they were averaged to form a single effect size.

^c^positive values indicate that faces manipulated to have larger FWHRs were judged as more threatening than those manipulated to have smaller FWHRs.

^d^After each face was rated individually, the authors also showed the pairs of faces side by side and had participants pick the one they believed to be tougher in a physical fight. Here, we use the D from the ratings in which each face of the pair was rated individually and then each image was compared to its counter-face, given such individual ratings provide more data unique to each facial identity. ^e^The authors also conducted a study wherein they had the individual faces, which were used to make the high and low FWHR composites, rated individually. The authors reported the correlation between the FWHR and the judgements and also reported the t-test value when the faces with high FWHRs were compared to those with smaller FWHRs. Given the second analysis utilizes less data than the first, we only used the first, which was included in the meta-analysis examining the link between the FWHR and perceptions of threat among studies that used a correlational design.

S9 Table. Effects included in the analysis of the relationship between the FWHR and perceptions of dominance, restricted to studies using a correlational design and/or a continuum of faces with un-manipulated FWHRs.

| Study | Observers | | | |  | Stimuli | | | | | Judgement | Effect Size (*r*)^a^ |
| --- | --- | --- | --- | --- | --- | --- | --- | --- | --- | --- | --- | --- |
|  | M | W | National-ity | *M*_age_ |  | M | W | National-ity | *M*_age_ | Display Details |  |  |
| Alrajih & Ward, [49] | 4^b^ | 6^b^ | UK | 21.5 |  | 153^c^ |  | UK | ~52.5 | Colour, 79 CEOs 74 controls (matched on ethnicity, age, facial hair, glasses) | D | **.280** |
| Burton & Rule, [62], Study 3, (average of ratings from S2A and S2B)^g^ | 34^d^ | 46^d^ | USA | 35^e^ |  | 50^f^ | 69^f^ | Canada | 23 | B/W | D | **.290** |
| Carré, McCormick, & Mondloch, [19], Study 1 | 15 | 16 | Canada | 19.94 |  | 24 |  | USA | 19.08 | 2000 ms, B/W | D | **.540** |
| Hehman, Leitner, & Freeman, [55], Study 3 | 12 | 12 |  |  |  | 60 |  | USA | 45 | 10 white faces from each decade (20s-70s) | SP | **.720** |
| Mileva, Cowan, Cobey, Knowles, & Little, [13], Study 1 | 10 | 9 |  | 26.4 |  | 50 |  | UK | 20.6 |  | D | **.340** |
| Mileva, Cowan, Cobey, Knowles, & Little, [13], Study 1 | 10 | 9 |  | 26.4 |  |  | 50 | UK | 20.6 |  | D | -.110 |
| Re, Hunter, Coetzee, Tiddeman, Xiao, DeBruine, Jones, & Perrett, [63] | 11 | 11 | UK | 25.32 |  | 47 | 83 |  | 23.84 | Faces retrieved from [www.3d.sk](http://www.3d.sk). | L | -.040 |
| Valentine, Li, Penke, & Perrett, [47] | 11^h^ | 44^h^ | Germany | 19.6 |  | 77 |  | Germany | 26.5 |  | D | **.220** |

Effect sizes in bold were reported as significant in the corresponding manuscripts. D = dominance. SP = social power. L = leadership (e.g., “How good of a leader do you think this person is?”). USA = United States of America. UK = United Kingdom. Int = International sample.

^a^positive values indicate that individuals with larger FWHRs are judged as more dominant than individuals with lower FWHRs. ^b^Sample size was estimated as these values given 8 men and 12 women were reported to have made three of six rating types each. ^c^Although the stimuli was 93 control and 93 CEO faces, thirty-three (14 CEOs) faces were removed because they were recognized by participants.

^d^These values were derived based on the estimate that the sample was 42 percent male.

^e^This value was the mean age reported for Study 2A; there was no age reported for 2B.

^f^two target faces were excluded; the sex of the targets was unspecified so we report the number of faces before exclusion.

^g^We do not provide separate effects from studies 2A and 2B because the relationships with the FWHR were not reported in these individual studies; the relationship with the FWHR was only reported after the ratings from these studies were averaged.

^h^The sample included one observer of an unknown sex. We only report the *n* for the number of participants of a known sex.

S10 Table. Effects included in the analysis of the relationship between the FWHR and perceptions of attractiveness, restricted to studies using a correlational design and/or a continuum of faces with un-manipulated FWHRs.

| Study | Observers | | | |  | Stimuli | | | | | Judgement^a^ | Effect Size (*r*)^b^ |
| --- | --- | --- | --- | --- | --- | --- | --- | --- | --- | --- | --- | --- |
|  | Men | Women | Nationality | *M*_age_ |  | Men | Women | Nationality | *M*_age_ | Display Details |  |  |
| Alrajih & Ward, [49] | 4^c^ | 6^c^ | UK | 21.5 |  | 153^d^ |  | UK | ~52.5 | Colour, 79 CEOs (m actual age = 52.5) and 74 controls (matched on ethnicity, age, facial hair, glasses) | ATT | -.100 |
| Boshyan et al., [18] Study 2 | 8 | 8 | USA | 75.6 |  | 24 |  | USA | 19.08 | 3000 ms, B/W | ATT | -.320 |
| Boshyan, et al., [18] Study 2 | 8 | 8 | USA | 18.8 |  | 24 |  | USA | 19.08 | 3000 ms, B/W | ATT | **-.470** |
| Carré, McCormick, & Mondloch, [19], Study 1 | 15 | 16 | Canada | 19.94 |  | 24 |  | USA | 19.08 | 2000 ms, B/W | ATT | -.224 |
| Geniole & McCormick, [20], Study 1 |  | 30 | Canada | 19.43 |  | 25 |  | Canada | 19.52 | 1000 ms, B/W | ATT: ***r* = -.390**); ST: ***r* = -.440**; LT: ***r* = -.470** | -.433^ƚ^ |
| Geniole & McCormick, [20], Study 1 |  | 30 | Canada | 19.43 |  | 54 |  | Canada | 20.32 | 1000 ms, Facegen, B/W | ATT: ***r* = -.700**; ST: ***r* = -.690**; LT: ***r* = -.770** | -.720^ƚ^ |
| Geniole & McCormick, [20], Study 2 |  | 26 | Canada | 20.69 |  | 54 |  | Canada | 20.32 | 1000 ms, Facegen, B/W | ATT | **-.550** |
| Geniole & McCormick, [21] | 22 | 34 | Canada | 19.89 |  | 25 |  |  |  | B/W, non-bearded faces^e^ | ATT | .330 |
| Geniole, Keyes, Mondloch, Carré, & McCormick, [22], Study 1 | 10 | 10 | Canada | 20.63 |  | 24 |  | USA | 19.08 | 2000 ms, B/W | ATT | -.240 |
| Geniole, Keyes, Mondloch, Carré, & McCormick, [22], Study 1 | 10 | 10 | Canada | 20.63 |  |  | 31 | USA | 18.87 | 2000 ms, B/W | ATT | -.010 |
| Geniole, Keyes, Mondloch, Carré, & McCormick, [22], Study 2 | 10 | 10 | Canada | 22.3 |  |  | 31 | USA | 18.87 | 2000 ms, B/W | ATT | .060 |
| Haselhuhn, Wong, Ormiston, Inesi, & Galinsky, [29], Study 4 | 2 | 2 | USA |  |  | 107 |  | USA |  |  | ATT | -.080 |
| Stirrat & Perrett, [34], Study 2 | 17 | 24 | UK | 21 |  | 67 |  | UK | 20.8 |  | ATT | **-.320** |
| Valentine, Li, Penke, & Perrett, [47] |  | 15 | Germany | 22.67 |  | 78 |  | Germany | 26.5 |  | ATT^f^ | -.180 |

Effect sizes in bold were reported as significant in the corresponding manuscripts.

^ƚ^Effect size represents an average for which we did not determine statistical significance. ATT = Attractiveness or unattractiveness (reversed). ST = Short-term desirability. LT = Long-term desirability. USA = United States of America. UK = United Kingdom. Int = International sample.

^a^If multiple effects are provided in a given cell, they were averaged to form a single effect size.

^b^positive values indicate that individuals with larger FWHRs are judged as more attractive than individuals with smaller FWHRs. ^c^Sample size of participants based on 8 men, 12 women, who only made three rating types each. Given 6 ratings total, this sample size was estimated to be 4 men and 6 women for each rating.

^d^Although the stimuli was 93 control and 93 CEO faces, thirty-three (14 CEOs) faces were removed because they were recognized by participants.

^e^In this manuscript the authors also examined the relationship between the FWHR and judgements of attractiveness in bearded versions of the same male faces (*r* = .25, *p* = .23). We chose to only include effects from the non-bearded versions of the faces given most perceptions studies involving the FWHR have used non-bearded faces as stimuli. We note that averaging the effect sizes, however, leads to the same mean weighted effect size in the final analysis (*k* = 14, $\bar{r}$ = -.26, *95% CI* = -.40 to -.11, *p* = .001; *Q*_13_ = 49.59, *p* < .0001).

^f^Some participants also speed-dated the men and, afterwards, provided ratings of interest in the men for short- and long-term relationships. Data from these social judgements were not included given that these effects likely reflect impressions based on social interactions rather than the facial structure of the men.

S11 Table. Effects included in the analysis on the relationship between the FWHR and BMI.

| Study | Sample Size | | Nationality of Sample | *M*_age_ | Effect Size (*r*)^a^ |
| --- | --- | --- | --- | --- | --- |
|  | Men | Women |  |  |  |
| Coetzee, Chen, Perrett, & Stephen, [64]^b^, Study 1, Caucasian Set A |  | 42 | UK | 20.9 | **.480** |
| Coetzee, Chen, Perrett, & Stephen, [64]^b^, Study 1, Caucasian Set B |  | 52 | UK | 19.9 | **.390** |
| Coetzee, Chen, Perrett, & Stephen, [64]^b^, Study 1, African Set A |  | 51 | South Africa | 19.8 | .270 |
| Coetzee, Chen, Perrett, & Stephen, [64]^b^, Study 1, African Set B |  | 48 | South Africa | 19.6 | **.330** |
| Coetzee, Chen, Perrett, & Stephen, [64]^b^, Study 1, Caucasian Set A | 41 |  | UK | 21.3 | **.330** |
| Coetzee, Chen, Perrett, & Stephen, [64]^b^, Study 1, Caucasian Set B | 54 |  | UK | 20.4 | .120 |
| Coetzee, Chen, Perrett, & Stephen, [64]^b^, Study 1, African Set A | 45 |  | South Africa | 21.2 | .150 |
| Coetzee, Chen, Perrett, & Stephen, [64]^b^, Study 1, African Set B | 47 |  | South Africa | 19.9 | .100 |
| Lefevre, Lewis, Bates, Dzhelyova, Coetzee, Deary, & Perrett, [12], Sample 1 | 46 | 99 | UK | 20.22 | **.270** |
| Lefevre, Lewis, Bates, Dzhelyova, Coetzee, Deary, & Perrett, [12], Sample 2 | 137 | 169 | UK | 83 | **.230** |
| Lefevre, Lewis, Bates, Dzhelyova, Coetzee, Deary, & Perrett, [12], Sample 3 | 91 | 98 | UK | 20.34 | **.400** |
| Lefevre, Lewis, Bates, Dzhelyova, Coetzee, Deary, & Perrett, [12], Sample 4 | 108 | 110 | South Africa | 20.22 | **.230** |
| Kramer, Jones, & Ward, [10], Study 3 |  | 105 | UK | 23.5 | **.430** |
| Kramer, Jones, & Ward, [10], Study 3 | 75 |  | UK | 23.5 | **.520** |
| Lefevre, Lewis, Perrett, & Penke, [42], Sample 1, from supplementary data | 188 |  | Germany | 33.6 | **.345** |
| Lefevre, Lewis, Perrett, & Penke, [42], Sample 2, from supplementary data | 76 |  | Germany | 20.5 | **.248**^c^ |
| Mayew, [65], supplementary data | 125 |  | Japan | 28.38 | **.179**^d^ |
| Loehr & O’Hara, [44], from Dryad dataset, subset of sample | 60 |  | Finland | 27.18 | .232 |
| Skorska, Geniole, Vrysen, McCormick, & Bogaert, [15] | 204 |  | Canada | 25.97 | **.387** |
| Skorska, Geniole, Vrysen, McCormick, & Bogaert, [15] |  | 186 | Canada | 22.62 | **.412** |
| Lefevre, Etchells, Howell, Clark, & Penton-Voak, [11] | 54 | 49 | UK | 21.59 | .090 |
| Welker, Goetz, & Carré, [66] | 146 |  | USA | 20.64 | **.40** |

*Notes:* Effect sizes in bold were reported as significant in the corresponding manuscripts. UK = United Kingdom. USA = United States of America. ^a^positive values indicate that individuals with larger FWHRs have higher BMI than individuals with lower FWHRs. ^b^These authors also provide results based on a meta-analysis of all of the effect sizes reported in their manuscript. Here, we only include the individual effect sizes. ^c^Because there was some ambiguity in the number of participants in the analysis reported in the paper, we used the author’s supplementary data set included with their manuscript to calculate this effect size. ^d^The data sheet from which we derived this effect size had two entries for some players (information on BMI for two seasons). We only used data for the first season for such players.

**References for Supplementary Tables**

1. Carré JM, McCormick CM. In your face: facial metrics predict aggressive behaviour in the laboratory and in varsity and professional hockey players. Proc Roy Soc B. 2008; 275: 2651–2656. doi:10.1098/rspb.2008.0873

2. Carré JM, Murphy KR, Hariri AR. What lies beneath the face of aggression? Soc Cogn Affect Neurosci. 2013;8: 224–229. doi:10.1093/scan/nsr096

3. Geniole SN, Keyes AE, Carré JM, McCormick CM. Fearless dominance mediates the relationship between the facial width-to-height ratio and willingness to cheat. Pers Individ Dif. 2014; 57: 59–64. doi:10.1016/j.paid.2013.09.023

4. Goetz SM, Shattuck KS, Miller RM, Campbell JA, Lozoya E, Weisfeld GE, Carré JM. Social status moderates the relationship between facial structure and aggression. Psychol Sci. 2013; 24: 2329–2334. doi:10.1177/0956797613493294

5. Gómez-Valdés J. et al. Lack of support for the association between facial shape and aggression: a reappraisal based on a worldwide population genetics perspective. PLOS ONE. 2013; 8: e52317. doi:10.1371/journal.pone.0052317

6. Haselhuhn MP, Wong EM. Bad to the bone: facial structure predicts unethical behaviour. Proc Roy Soc B. 2012; 279: 571–576. doi:10.1098/rspb.2011.1193

7. Hehman E, Leitner JB, Gaertner SL. Enhancing static facial features increases intimidation. J Exp Soc Psychol. 2013; 49: 747–754. doi:10.1016/j.jesp.2013.02.015

8. Huh H. Digit ratios, but not facial width-to-height ratios, are associated with the priority placed on attending to faces versus bodies. Pers Individ Dif. 2013; 54: 133–136. doi:10.1016/j.paid.2012.08.032

9. Huh H, Yi D, Zhu H. Facial width-to-height ratio and celebrity endorsements. Pers Individ Dif. 2014; 68: 43–47. doi:10.1016/j.paid.2014.03.044

10. Kramer RSS, Jones AL, Ward R. A lack of sexual dimorphism in width-to-height ratio in white European faces using 2D photographs, 3D scans, and anthropometry. PLOS ONE. 2012; 7: e42705. doi:10.1371/journal.pone.0042705

11. Lefevre CE, Etchells PJ, Howell EC, Clark AP, Penton-Voak IS. Facial width-to-height ratio predicts self-reported dominance and aggression in males and females, but a measure of masculinity does not. Biol Lett. 2014; 10: 20140729. doi:10.1098/rsbl.2014.0729

12. Lefevre CE, Lewis GJ, Bates TC, Dzhelyova M, Coetzee V, Deary IJ, Perrett, DI. No evidence for sexual dimorphism of facial width-to-height ratio in four large adult samples. Evol Hum Behav. 2012; 33: 623–627. doi:10.1016/j.evolhumbehav.2012.03.002

13. Mileva VR, Cowan ML, Cobey K D, Knowles KK, Little AC. In the face of dominance: Self-perceived and other-perceived dominance are positively associated with facial-width-to-height ratio in men. Pers Individ Diff. 2010; 69: 115–118. doi:10.1016/j.paid.2014.05.019

14. Sanchez-Pages S, Rodriguez-Ruiz C., Turiegano E. Facial masculinity : how the choice of measurement method enables to detect its influence on behaviour. PLOS ONE. 2014; 9: e112157. doi:10.1371/journal.pone.0112157

15. Skorska MN, Geniole SN, Vrysen BM, McCormick CM, Bogaert AF. Facial structure predicts sexual orientation in both men and women. Arch Sex Behav. in press; doi:10.1007/s10508-014-0454-4

16. Stirrat M, Stulp G, Pollet TV. Male facial width is associated with death by contact violence: Narrow-faced males are more likely to die from contact violence. Evol Hum Behav. 2012; 33: 551–556. doi:10.1016/j.evolhumbehav.2012.02.002

17. Weston EM, Friday AE, Liò P. Biometric evidence that sexual selection has shaped the hominin face. PLOS ONE. 2007; 2:e710. doi:10.1371/journal.pone.0000710

18. Boshyan J, Zebrowitz LA, Franklin RG, McCormick CM, Carré JM. Age similarities in recognizing threat from faces and diagnostic cues. J Gerontol B. Psychol Sci Soc Sci. , 2014; 69: 710-718. doi:10.1093/geronb/gbt054

19. Carré JM, McCormick CM. Mondloch CJ Facial structure is a reliable cue of aggressive behavior. Psychol Sci. 2009; 20: 1194–1198. doi:10.1111/j.1467-9280.2009.02423.x

20. Geniole SN, McCormick CM. Taking control of aggression: Perceptions of aggression suppress the link between perceptions of facial masculinity and attractiveness. Evol Psychol. 2013; 11: 1027–1043.

21. Geniole SN, McCormick CM. Facing our ancestors: Judgements of aggression are consistent and related to the facial width-to-height ratio in men irrespective of beards. Evol Hum Behav. in press; doi:10.1016/j.evolhumbehav.2014.12.005

22. Geniole SN, Keyes AE, Mondloch CJ, Carré JM, McCormick CM. Facing aggression: cues differ for female versus male faces. PLOS ONE. 2012; 7: e30366. doi:10.1371/journal.pone.0030366

23. Cherek DR. Psychopharmacology effects of smoking different doses of nicotine on human aggressive behavior. Psychopharmacology. 1981; 75, 339–345.

24. Buss AH, Perry M. The Aggression Questionnaire. J Pers Soc Psychol. 1992; 63: 452–459.

25. Deaner RO, Goetz SMM, Shattuck K, Schnotala T. Body weight, not facial width-to-height ratio, predicts aggression in pro hockey players. J Res Pers. 2012; 46: 235–238. doi:10.1016/j.jrp.2012.01.005

26. Efferson C, Vogt S. Viewing men’s faces does not lead to accurate predictions of trustworthiness. Sci Rep. 2013; 3: 1047. doi:10.1038/srep01047

27. Karp R, Gold D, Tan M. Bullard Houses. Cambridge, MA: Harvard University, Harvard Law School, Program on Negotiation. 2006.

28. Haselhuhn, M. P., Wong, E. M. & Ormiston, M. E. 2013 Self-fulfilling prophecies as a link between men’s facial width-to-height ratio and behavior. PLOS ONE 2013; 8: e72259. doi:10.1371/journal.pone.0072259

29. Haselhuhn MP, Wong EM, Ormiston ME, Inesi ME, Galinsky AD. Negotiating face-to-face: Men’s facial structure predicts negotiation performance. Leadersh. Q. 2014; 25, 835–845. doi:10.1016/j.leaqua.2013.12.003

30. Hehman E, Leitner JB, Deegan MP, Gaertner SL. Facial structure is indicative of explicit support for prejudicial beliefs. Psychol Sci. 2013; 24: 289–296. doi:10.1177/0956797612451467

31. Jia Y, van Lent L, Zeng,Y. Masculinity, testosterone, and financial misreporting. J Account Res. 2014; 52: 1195–1246. doi:10.1111/1475-679X.12065

32. Özener B. Facial width-to-height ratio in a Turkish population is not sexually dimorphic and is unrelated to aggressive behavior. Evol Hum Behav. 2012; 33: 169–173. doi:10.1016/j.evolhumbehav.2011.08.001

33. Buss AH, Warren WL. The aggression questionnaire manual. Los Angeles, CA: Western Psychological Services; 2000.

34. Stirrat M, Perrett DI. Valid facial cues to cooperation and trust: male facial width and trustworthiness. Psychol Sci. 2010; 21: 349–354. doi:10.1177/0956797610362647

35. Stirrat M, Perrett DI. Face structure predicts cooperation: men with wider faces are more generous to their in-group when out-group competition is salient. Psychol Sci. 2012; 23: 718–722. doi:10.1177/0956797611435133

36. Třebický V, Fialová J, Kleisner K, Roberts SC, Little AC, Havlíček J. Further evidence for links between facial width ‐ to ‐ height ratio and fighting success: commentary on Zilioli et al . ( 2014 ). Aggress Behav. in press; doi:10.1002/ab.21559

37. Welker KM, Goetz SM., Galicia S, Liphardt J, Carré JM. An examination of the associations between facial structure, aggressive behavior, and performance in the 2010 world cup association football players. Adapt Hum Behav Physiol. 2014; 1: 1–13. doi:10.1007/s40750-014-0003-3

38. Zilioli S, Sell AN, Stirrat M, Jagore J, Vickerman W, Watson NV. Face of a fighter: Bizygomatic width as a cue of formidability. Aggress Behav. in press; doi:10.1002/ab.21544

39. Goldberg LR, Johnson JA, Eber HW, Hogan R, Ashton MC, Cloninger CR, Gough HG. The international personality item pool and the future of public-domain personality measures. J Res Pers. 2006; 40: 84–96. doi:10.1016/j.jrp.2005.08.007

40. Lilienfeld SO, Widows MR. Pychopathic Personality Inventory- Revised: Professional Manual. Lutz, FL: Psychological Assessment Resources; 2005.

41. Anderson C, Galinsky AD. Power, optimism, and risk-taking. Eur J Soc Psychol. 2006; 36: 511–536. doi:10.1002/ejsp.324

42. Lefevre CE, Lewis GJ, Perrett DI, Penke L. Telling facial metrics: Facial width is associated with testosterone levels in men. Evol Hum Behav. 2013; 34: 273–279. doi:10.1016/j.evolhumbehav.2013.03.005

43. Lewis GJ, Lefevre CE, Bates TC. Facial width-to-height ratio predicts achievement drive in US presidents. Pers Individ Diff. 2012; 52: 855–857. doi:10.1016/j.paid.2011.12.030

44. Loehr J, O’Hara RBO. Facial morphology predicts male fitness and rank but not survival in Second World War Finnish soldiers. Biol Lett. 2013; 9: 20130049. doi:10.1098/rsbl.2013.0049

45. Goldberg L. A broad-bandwidth, public domain, personality inventory measuring the lower-level facets of several five-factor models. In Personality Psychology in Europe (eds I. Mervielde I. J. Deary F. De Fruyt & F. Ostendorf), pp. 7–28. Tillburg: Tilburg University Press; 1999.

46. Cheng JT, TracyJL, Henrich J. Pride, personality, and the evolutionary foundations of human social status. Evol Hum Behav. 2010; 31: 334–347. doi:10.1016/j.evolhumbehav.2010.02.004.

47. Valentine KA, Li NP, Penke L, Perrett DI. Judging a man by the width of his face: the role of facial ratios and dominance in mate choice at speed-dating events. Psychol Sci. 2014; 25: 806–811. doi:10.1177/0956797613511823

48. Ostendorf F. Measuring interpersonal behavior with the German Interpersonal Adjective Scales. In Personality and temperament: Genetics, evolution, and structure (eds R. Riemann F. M. Spinath & F. Ostendorf), pp. 232–260. Lengerich, Germany: Pabst.; 2001.

49. Alrajih S Ward J. Increased facial width-to-height ratio and perceived dominance in the faces of the UK’s leading business leaders. Br J Psychol. 2014; 105: 153–161. doi:10.1111/bjop.12035

50. Wong EM, Ormiston ME, Haselhuhn MP. A face only an investor could love: CEOs’ facial structure predicts their firms' financial performance. Psychol Sci. 2011; 22: 1478–1483. doi:10.1177/0956797611418838

51. Mayew WJ, Parsons CA, Venkatachalam M. Voice pitch and the labor market success of male chief executive officers. Evol Hum Behav. 2013; 34: 243–248. doi:10.1016/j.evolhumbehav.2013.03.001

52. Tsujimura H, Banissy MJ. Human face structure correlates with professional baseball performance: insights from professional Japanese baseball players. Biol Lett. 2013; 9: 20130140. doi:10.1098/rsbl.2013.0140

53. Carré JM, Morrissey MD, Mondloch CJ, McCormick, CM. Estimating aggression from emotionally neutral faces: Which facial cues are diagnostic? Perception. 2010; 39: 356–377. doi:10.1068/p6543

54. Geniole SN, Molnar DS, Carré JM, McCormick CM. The facial width-to-height ratio shares stronger links with judgments of aggression than with judgments of trustworthiness. J Exp Psychol Hum Percept Perform. 2014; 30: 1526-1541. doi:10.1037/a0036732

55. Hehman E, Leitner JB, Freeman JB. The face – time continuum: lifespan changes in facial width-to-height ratio impact aging-associated perceptions. Personal Soc Psychol Bull. 2014; 40: 1624–1636. doi:10.1177/0146167214552791

56. Kleisner K, Priplatova L, Frost P, Flegr J. Trustworthy-looking face meets brown eyes. PLOS ONE 2013; 8: 10.1371/journal.pone.0053285. doi:10.1371/journal.pone.0053285

57. Short LA, Mondloch CJ, McCormick CM, Carré JM, Ma R, Fu G, Lee K. Detection of propensity for aggression based on facial structure irrespective of face race. Evol Hum Behav. 2012; 33: 121–129. doi:10.1016/j.evolhumbehav.2011.07.002

58. Marsh, A. A., Cardinale, E. M., Chentsova-Dutton, Y. E., Grossman, M. R. & Krumpos, K. A. Power Plays: Expressive Mimicry of Valid Agonistic Cues. Soc Psychol Personal Sci. 2014; 5, 684–690. doi:10.1177/1948550613519684

59. Bashir NY, Rule NO. Shopping under the influence: nonverbal appearance-based communicator cues affect consumer judgments. Psychol Mark. 2014; 31: 539–548. doi:10.1002/mar

60. Lefevre CE, Lewis GJ. Perceiving aggression from facial structure: further evidence for a positive association with facial width-to-height ratio and masculinity, but not for moderation by self-reported dominance. 2013; 28: 530-537. doi:10.1002/per

61. Wang Y, Geigel J, Herbert A. Reading personality: Avatar vs. human faces. Proc - 2013 Hum Assoc Conf Affect Comput Intell Interact. ACII 2013; 479–484. doi:10.1109/ACII.2013.85

62. Burton CM, Rule NO. Judgments of height from faces are informed by dominance and facial maturity. Soc Cogn. 2013; 31: 672–685. doi:10.1521/soco.2013.31.6.672

63. Re DE, Hunter DW, Coetzee V, Tiddeman BP, Xiao D, DeBruine LM, Jones BC, Perrett, DI. Looking like a leader-facial shape predicts perceived height and leadership ability. PLOS ONE. 2013; 8: e80957. doi:10.1371/journal.pone.0080957

64. Coetzee V, Chen J, Perrett, DI, Stephen, ID. Deciphering faces: quantifiable visual cues to weight. Perception 2010; 39: 51–61. doi:10.1068/p6560

65. Mayew WJ. Reassessing the association between facial structure and baseball performance: a comment on Tsujimura & Banissy (2013). Biol Lett. 2013; 9: 538. doi:10.1098/rsbl.2013.0538

66. Welker KM, Goetz, SM, Carré, JM Perceived and experimentally manipulated status moderate the relationship between facial structure and risk-taking. Evol Hum Behav. in press. doi: 10.1016/j.evolhumbehav.2015.03.006
