# Supplementary figures and images for: Evidence from Meta-Analyses of the Facial Width-to-Height Ratio as an Evolved Cue of Threat
Source: PLoS One. 2015 Jul 16;10(7):e0132726. doi: 10.1371/journal.pone.0132726 (PMC4504483; doi:10.1371/journal.pone.0132726)

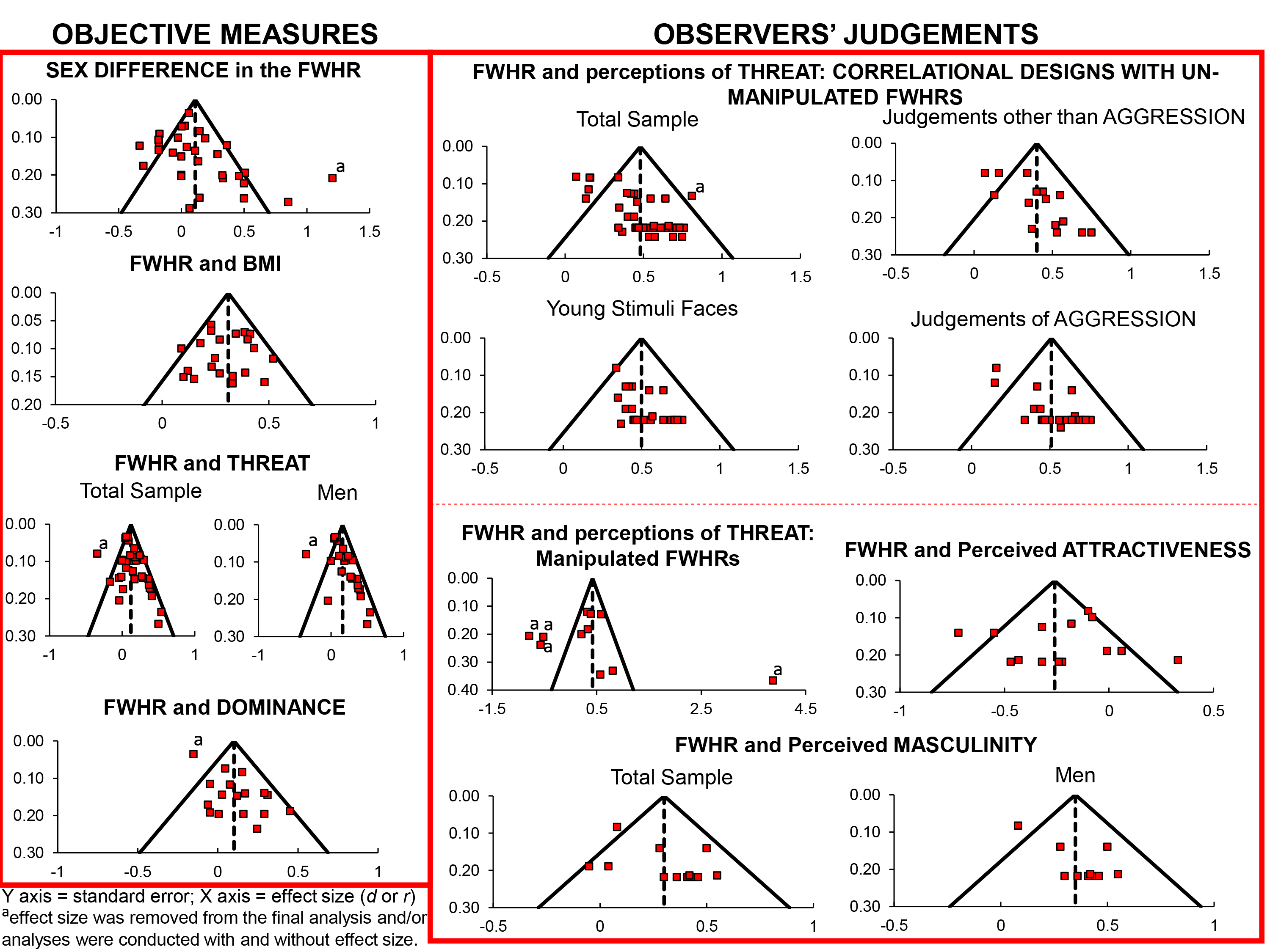

Supplement: S1 Fig — (TIF) [file pone.0132726.s004.tif]
